# Evaluating Sustainable Feed Alternatives in *Sparus aurata*: How Alternative Proteins and Oils Maintain EPA+DHA Content and Improve Human Health Lipid Indices

**DOI:** 10.3390/foods15101762

**Published:** 2026-05-16

**Authors:** Esther Sendra, Isabel Casanova-Martínez, Marcos Rodríguez-Estrada, Josep Àlvar Calduch-Giner, Jaume Pérez-Sánchez, Marina Cano-Lamadrid

**Affiliations:** 1 Instituto de Investigación e Innovación Agroalimentaria y Agroambiental (CIAGRO-UMH), Universidad Miguel Hernández, Carretera de Beniel, Km 3.2, 03312 Orihuela, Alicante, Spain; icasanova@umh.es (I.C.-M.); marcos.rodrigueze@umh.es (M.R.-E.); marina.canol@umh.es (M.C.-L.); 2 Fish Nutrigenomics and Integrative Biology Group, Institute of Aquaculture Torre de la Sal (IATS, CSIC), 12595 Ribera de Cabanes, Castellón, Spain; j.calduch@csic.es (J.À.C.-G.); jaime.perez.sanchez@csic.es (J.P.-S.)

**Keywords:** DHA (docosahexaenoic acid), EPA (eicosapentaenoic acid), alternative protein sources, insect meal, fish oil, health claim, sustainable aquaculture

## Abstract

This study evaluated the effects of alternative feed formulations on the proximate composition and lipid quality of gilthead sea bream (*Sparus aurata*) in a long-term feeding trial (May 2022–September 2023). Three isoenergetic and isoproteic diets were tested in replicate tanks: a fishmeal-based control (CTRL), a processed animal protein–based diet (PAP), and a diet including insect meal and microalgae oil (ALT). Diet pellet sizes were adapted to the fishes’ developmental stage. Proximate composition and fatty acid profiles were assessed in feed and in fish fillets, with 20 fish analyzed per dietary treatment. The human health lipid indices of the fillets were calculated. Virtual diets were reconstructed to estimate theoretical fatty acid intake across growth, based on feed composition and consumption. Partial least squares discriminant analysis (PLS-DA) revealed distinct clustering by diet. Fillets from all diets met European Food Safety Authority criteria for being high in omega-3 fatty acids, with some variation in EPA and DHA concentrations among formulations. The ALT diet showed a 15% higher EPA+DHA content and the greatest fish lipid quality (FLQ) values, even having the lowest polyunsaturated fatty acids (PUFA) intake from feed, partly due to its elevated lauric acid (C12:0) content, which may contribute to rapid energy mobilization and omega-3 preservation. PAP-fed fish showed the most balanced PUFA/SFA and n6/n3 ratios. These findings demonstrate the viability of sustainable feed alternatives for maintaining nutritional quality in gilthead sea bream, supporting aquaculture sustainability without compromising nutritional value.

## 1. Introduction

Aquaculture has emerged as a fundamental solution to meet the growing global demand for seafood products, playing a key role in addressing the increasing demand for fish protein in the context of declining wild fish stocks. Gilthead sea bream (*Sparus aurata*) is one of the most economically important species in Mediterranean aquaculture due to its great adaptability to intensive culture conditions in both ponds and cages. In recent decades, its production has grown continuously, exceeding 200,000 tons in 2020, driven by advances in breeding, nutrition, and the development of aquaculture feeds [1]. As aquaculture intensifies, fish nutrition remains a critical aspect of improving growth performance, health status, and product quality, all of which are closely related to diet composition and nutrient availability [2]. Fundamental to the success of this sector is the formulation of nutritionally balanced feeds, in which fatty acids play a key role in supporting fish growth, health, and overall product quality.

Fatty acids, especially long-chain polyunsaturated fatty acids (LC-PUFAs), omega-3 fatty acids such as eicosapentaenoic acid (EPA; 20:5n3), and docosahexaenoic acid (DHA; 22:6n3), are indispensable for cellular integrity, immune function, and metabolic processes in fish [3]. Carnivorous fish species such as gilthead sea bream have dietary requirements for essential fatty acids (EFAs), especially LC-PUFAs (n3 and n6 series with 20 or more carbons) EPA, DHA, and arachidonic acid (20:4n-6). These fatty acids not only contribute to fish health and growth, but also enhance the nutritional value of aquaculture products for human consumption, particularly due to the well-established cardiovascular and cognitive benefits of EPA and DHA [4,5,6,7]. Despite this recognized importance, there is still limited knowledge of how alternative feed formulations, especially those that reduce or replace marine ingredients, affect the deposition and balance of these key fatty acids in farmed fish. Recent evidence confirms that the fatty acid composition of gilthead sea bream is strongly influenced by both dietary inputs—such as fishmeal, algae, or insect-based formulations—and the production system, with notable differences between wild and farmed fish. In particular, farmed gilthead sea bream exhibit higher levels of omega-3 fatty acids (DHA and EPA) due to lipid-rich aquaculture diets, whereas wild individuals show lower lipid content and a distinct metabolite profile shaped by environmental adaptation and natural feeding regimes [8].

Given its physiological importance both for fish and human health, ensuring an adequate supply of these essential lipids in aquafeeds is critical for the sustainable development of aquaculture. Traditionally, fishmeal (FM) and fish oil (FO) have been the main sources of essential fatty acids in aquafeeds, considering both the biological quality of the protein and its digestibility. However, their continued use poses significant challenges, including sustainability concerns linked to overfishing, fluctuating costs, and environmental impact [9,10]. These problems have prompted extensive research into alternative ingredients that could replace FM and FO without compromising feed efficiency or the nutritional quality of the fish. At the fish farm level, FM substitution could have a positive effect on production costs [11]. It is important to note that the elimination of FO from aquaculture diets is challenging. If it is removed, the DHA content in farmed fish decreases, and given the scarcity of fish stocks, the absence of alternatives to maintain DHA would reduce the overall omega-3 content and compromise fish quality. Nevertheless, to address these challenges, researchers have studied a wide range of alternative feed ingredients, such as plant proteins, microalgae, and single-cell proteins, as potential substitutes for FM and FO.

Plant-based proteins, such as soybean meal, pea protein, and wheat gluten, have shown promising results in terms of protein digestibility and utilization in various fish species [12]. Although early studies highlighted limitations of plant proteins due to anti-nutritional factors and amino acid imbalances [13], it has been demonstrated that these adverse effects can be mitigated through the use of specific feed additives [14,15]. Indeed, diets combining plant proteins with insect protein and microbial biomass, supplemented with gut health promoters, have shown promising results in gilthead sea bream, ensuring proper growth and health even under challenging conditions [15]. In addition, plant-derived oils lack the essential omega-3 polyunsaturated fatty acids necessary for optimal fish health and human nutrition, requiring further research into new lipid sources. Moreover, microalgae have emerged as a valuable lipid source, providing highly bioavailable omega-3 LC-PUFAs such as DHA in a sustainable and renewable form [16]. Dietary inputs significantly influence the lipid composition of farmed fish, making it crucial to understand how alternative feed formulations affect the profile of essential fatty acids such as EPA and DHA. Therefore, ensuring that sustainable feed alternatives preserve or even enhance the levels of these beneficial fatty acids is essential to meet consumer expectations regarding taste, texture, and nutritional value. The inclusion of alternative ingredients must therefore be evaluated not only for its impact on fish performance, but also for its effects on lipid composition and human health indices.

This study aims to evaluate the impact of two alternative protein–based diets on fish lipid quality in a long-term feeding trial. One diet was based on processed animal proteins and another on insect and algae sources, compared to a conventional fishmeal-based control, on the proximate composition, fatty acid profile, and human health lipid indices of gilthead sea bream. These formulations, in parallel experiments performed as part of a larger project, have demonstrated comparable growth performance under farming conditions [17,18,19]. Building on those findings, the present work focuses on lipid quality and health-promoting properties, offering valuable insights into the feasibility of sustainable aquaculture practices to maintain nutritional value and product quality.

## 2. Materials and Methods

### 2.1. Animal Ethics

All fish procedures were approved by the Ethics and Animal Welfare Committee of IATS, the CSIC Ethics Committee (permission 1295/2022), and Generalitat Valenciana (permission 2022-VSC-PEA-0230). They were carried out in the IATS’s registered aquaculture infrastructure facility (code ES120330001055) in accordance with the principles published in the European Animal Directive (2010/63/EU) and Spanish laws (Royal Decree RD53/2013) for the protection of animals used in scientific experiments.

### 2.2. Experimental Design

Three types of feed formulations (CTRL: fishmeal–based control diet, PAP: processed animal protein–based diet, and ALT: alternative protein–based diet, incorporating insect meal and microbial biomass) were tested. All diets were formulated to be isoenergetic and isoproteic, with adjustments in composition according to pellet size to meet the nutritional needs of gilthead sea bream. Feeds were manufactured by Sparos Lda (Olhão, Portugal) and produced in four pellet sizes (2 mm, 3 mm, 4.5 mm, 6 mm), corresponding to different developmental stages (Table S1). The CTRL diet reflected commercial formulations, combining FM, FO, and plant ingredients. The PAP diet replaced FM with poultry meal and plant proteins, whereas the ALT diet utilized insect protein and microbial biomass (Aminopro NT70) as substitutes for FM. FO inclusion ranged from 5.4% to 6.6%, and additional oils were incorporated to meet DHA and EPA requirements: salmon oil by-products (3–7%) in PAP and DHA-rich microalgae oil (0.4–0.9%) in ALT. Rapeseed oil was also included in all diets, except PAP, at the 2 mm pellet size.

**Figure 1:**
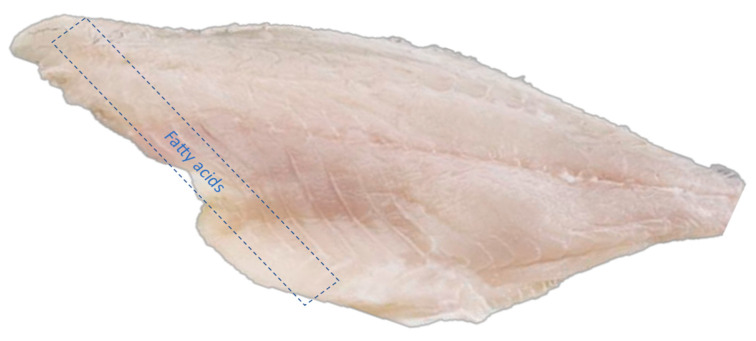
Fillet section selected for freeze-drying and analysis of the fatty acid profile.

Gilt head seabream (*Sparus aurata*) juveniles, with an initial body weight of 15 g, were reared in a continuous-flow system (3000 L tanks) from May 2022 to September 2023 at the IATS indoor experimental facilities, using three experimental diets under natural temperature and photoperiod conditions (40°5′ N; 0°10′ E). The fish were distributed into three dietary groups according to the experimental feeds: a control diet based on fishmeal (CTRL), a diet based on processed animal protein (PAP), and an alternative diet containing insect meal and microbial biomass (ALT). Each dietary group was assigned to two replicate tanks (six tanks in total). Fish were fed near to visual satiety using automated feeders, with pellet size adjusted to fish growth (2–6 mm), 3–7 days per week, depending on the season and fish size. The water oxygen concentration was maintained above 75% saturation, and stocking densities were kept below 16 kg/m^3^. At the end of the trial, when fish reached 800 g (September 2023), 10 individuals per tank were randomly sampled (*n* = 20 fish per dietary group: CTRL, PAP, ALT). The animals were killed by concussion and subsequent decapitation, and the same section of the right fillet was freeze-dried and stored at −80 °C before analyzing the fatty acid profiles (Figure 1). Proximate composition was analyzed in a homogenate of the fillet. Feed samples (12) were analyzed in triplicate, and fish samples (60) were analyzed in duplicate.

### 2.3. Proximal Composition Analysis

For the analysis of the feed (CTRL, PAP, ALT), the parameters of fat, protein, crude fiber, ash, organic matter, and dry matter were evaluated to determine the nutritional composition. In the case of fish, fat, protein, ash, and dry matter were determined.

#### 2.3.1. Crude Fat

The procedure for determining crude fat begins by drying the samples in aluminum containers at 60 °C and cooling them in a desiccator. Two grams of sample were weighed into cellulose cartridges and extracted using ethyl ether (C_2_H_5_)_2_O in a DET-GRAS instrument (P Selecta, Barcelona, Spain). After extraction, the samples were dried again at 60 °C for 24 h and weighed to determine the crude fat content [20].

#### 2.3.2. Fiber

The samples were dried, ground (1 mm), and degreased with petroleum ether. Approximately 1 g was placed in filter bags and subjected to sequential digestion with sulfuric acid (0.255 N) and sodium hydroxide (0.313 N) at 100 °C (30 min each), with intermediate rinses in the AKNOM200 analyzer (ANKOM Technology, Macedon, NY, USA). Subsequently, they were rinsed with methanol, dried, and calcined at 550 °C, and the ash content was corrected to obtain crude fiber [21].

#### 2.3.3. Protein

First, 0.1 g of the sample was digested with H_2_SO_4_ and catalyst tablets (Kjel-tabs) at 420 °C for 45 min. After digestion, the analysis was performed using an automated Kjeldahl system, and the percentage of nitrogen or protein was calculated using conversion factors [22].

#### 2.3.4. Ash, Organic Matter, and Dry Matter

The samples were calcined at 550 °C, cooled, and dried at 103 °C prior to weighing. Organic matter was calculated as the difference between dry matter and ash. Dry matter was determined by drying the sample at 102 ± 2 °C until a constant weight was reached [23,24].

### 2.4. Fatty Acid Profile

Freeze-dried samples were methylated following a direct extraction-methylation method with a first basic step with sodium methoxide, followed by an acid step with boron trifluoride 14% in methanol, as described by Trigueros and Sendra [25]. A gas chromatograph coupled to a flame ionization detector (GC-FID) was used for detection and semi-quantification (percentage of each fatty acid in the total fatty acid profile), with the specific equipment parameters as described in Table S2. Fatty acid ratios and human health indices were calculated following formulas compiled previously [26,27] and are detailed in Table S3.

### 2.5. Virtual Diet Calculation

To estimate the theoretical cumulative fatty acid exposure of gilthead sea bream throughout the production cycle, virtual diets were reconstructed for each dietary treatment (CTRL, PAP, ALT). This approach is based on the concept that tissue fatty acid composition reflects long-term dietary intake in fish, particularly under commercial aquaculture feeding practices, where diets are sequentially adjusted in terms of protein, lipid, and energy content according to fish size and growth stage [28,29]. The virtual diet reconstruction was designed to reflect the sequential use of feeds with different pellet sizes and formulations that characterizes commercial aquaculture feeding practices and that was reproduced in the present long-term trial, accounting for the cumulative effect of differences in fatty acid composition among the various pellet sizes administered throughout the grow-out period. Each virtual diet model considered the specific fatty acid profile of every pellet size and its relative contribution to total dietary supply. Feed intake proportions were estimated at the tank level, based on recorded feed administration data and standard feeding adjustments according to fish size, temperature, and season. The percentage contribution of each pellet size to total feed intake was calculated from the duration of use of each granulometry and the corresponding feeding rates. The percentage intake of feed is shown in Figure S1.

Equation (1) was used to calculate the intake of each fatty acid by the fish.

(1) Intake of (fatty acid) = [% intake of 2 mm feed × concentration of (fatty acid) in 2 mm feed] + [% intake of 3 mm feed × concentration of (fatty acid) in 3 mm feed] + [% intake of 4.5 mm feed × concentration of (fatty acid) in 4.5 mm feed] + [% intake of 6 mm feed × concentration of (fatty acid) in 6 mm feed].

### 2.6. Statistical Analysis

The tank was considered the experimental unit, with individual fish subsampled (10 per tank) to capture within-tank biological variability. These sample sizes were based on standard practice in aquaculture nutrition trials and were selected to provide sufficient sensitivity to detect biologically relevant differences while maintaining experimental and ethical feasibility. Statistical analyses were performed using IBM SPSS Statistics 29.0.0.0 software. Non-parametric tests were applied, as a normality test was conducted, which determined that the variables analyzed did not follow a normal distribution. Therefore, to compare the differences between more than two independent groups, a non-parametric test was used, specifically the one-way Kruskal–Wallis test. This test evaluates whether statistically significant differences exist among the medians of the groups compared. When significant differences were detected, multiple post hoc comparisons were conducted using the stepwise step-down procedure to identify the specific groups in which such differences occurred. This approach reduces the risk of type I error associated with multiple comparisons. The level of statistical significance was set at *p* < 0.05. On the other hand, to examine detailed differences in fatty acid profiles among groups, partial least-squares discriminant analysis (PLS-DA) was carried out using EZinfo v3.0 (Umetrics, Umeå, Sweden). The quality of the PLS-DA model was assessed using the parameters R2Y (cum) and Q2Y (cum), which represent the model’s goodness of fit and prediction ability, respectively. The contribution of individual fatty acids to group separation was evaluated based on their variable importance in projection (VIP) values. A VIP score greater or equal to 1 was used as the threshold to identify discriminant variables within the PLS-DA model [30].

## 3. Results

### 3.1. Feed Proximate Composition and Essential and Major Fatty Acids

The proximate composition of the different fish feeds (Table 1) confirmed that all diets were formulated with broadly similar nutritional profiles. Although some differences were observed among formulations, most variations were associated with pellet size rather than the diet itself. Crude fat content spanned from 16.3% (PAP4.5) to 19.6% (ALT3), and protein from 43.2% (PAP6) to 51.0% (PAP2), with variations mainly linked to pellet size rather than major differences in overall composition. Fiber content ranged from 1.41% (CTRL2) to 2.51% (ALT4.5). Ash content varied between 5.88% (PAP4.5) and 8.59% (CTRL6), with the highest values in the CTRL diet, while organic matter content ranged from 84.9% (ALT6) to 89.0% (PAP3), with PAP generally showing the highest values and ALT the lowest. Dry matter content varied among formulations, fluctuating from 92.8% (ALT6) to 96.4% (CTRL3), with no consistent pattern across pellet sizes, although 6.0 mm feeds tended to have the lowest dry matter content.

MUFA was the most abundant group in all feeds. The essential and major fatty acids of the feed formulations are shown in Table 2, with the complete profiles detailed in the Supplementary Material (Table S4), expressed as the percentage of total fatty acids in the total profile. The saturated fatty acid (SFA) content fluctuated from 21.92% (PAP3) to 27.44% (ALT6), with ALT showing the highest overall levels across pellet sizes. MUFA varied between 39.85% (PAP2) and 46.94% (CTRL3), with CTRL generally showing the highest values, while PAP and ALT presented similar, lower ranges. The polyunsaturated fatty acid (PUFA) percentage ranged from 30.27% (CTRL3) to 34.17% (PAP3), with PAP having a slightly higher PUFA content than CTRL and ALT. EPA levels ranged from 5.40% (CTRL3) to 8.27% (PAP6), while DHA levels ranged between 2.75% (CTRL6) and 5.94% (ALT2). In both PAP and ALT, DHA tended to decrease with increasing pellet size, whereas this trend was less consistent in CTRL. Finally, differences in the sum of omega-3 fatty acids between granulometries and between formulations were moderate, consistent with the intended nutritional design.

### 3.2. Essential and Major Fatty Acids in Virtual Feed

**Figure 2:**
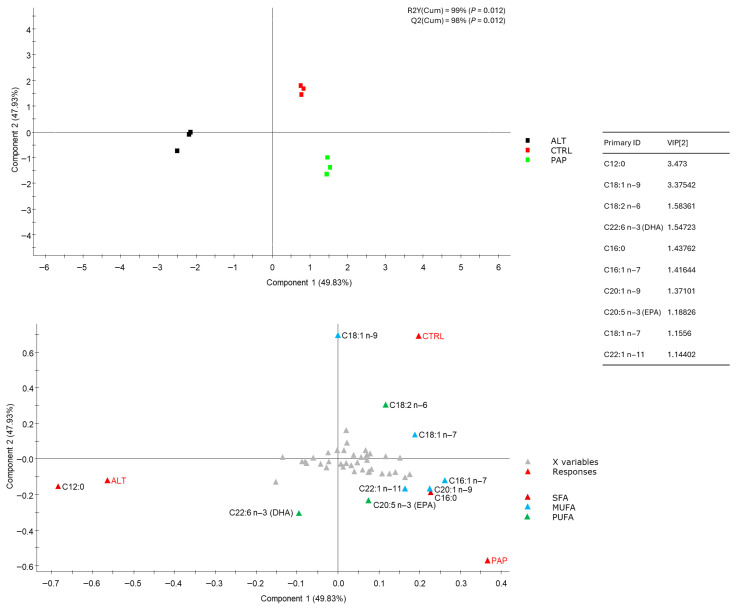
Two-dimensional representation of the distribution of virtual diet samples (n = 9) between the two first components of a partial least squares discriminant analysis (PLS-DA) model driving the separation of groups based on two components. The upper panel shows the classification of the samples by group. The lower panel (biplot) displays both the sample centroids and the explanatory variables (fatty acids), colored according to their class (SFA, MUFA, PUFA). Additionally, the table associated with the model lists the fatty acids with the greatest contribution to group separation (components 1 and 2) (VIP > 1), indicating their relative importance in the projection.

To provide an integrated view of the theoretical fatty acid supply throughout the grow-out period, a virtual feed was constructed, considering the whole profile of fatty acids of each granulometry (Table S4) and the estimated intake proportions (Figure S1). Figure 2 shows the two-dimensional representation of virtual diet samples (n = 9) based on the first two components of the partial least squares discriminant analysis (PLS-DA) model, which summarize the main sources of variation in the dataset. The analysis indicated three distinct clusters: ALT, CTRL, and PAP [variance explained, R2Y(Cum): 99%; variance predicted, Q2Y(Cum): 98%] (Figure 2 upper panel). Model validation and component contribution are detailed in Figure S2: *R*^2^*Y*^2,^ and *Q*^2^*Y* are both above 0.8, and their permutation *p*-value is 0.014, which is considered a highly robust and predictive model. Among the 50 identified fatty acids, those with VIP scores > 1 contributed most to group separation (Figure 2 lower panel). Variables with VIPs below 1 were colored in grey for clarity. CRTL and PAP were characterized by higher MUFA and PUFA contents, including EPA content, whereas ALT showed higher SFA (notably C12:0) and DHA levels.

### 3.3. Proximate Composition and Fatty Acid Profile of Gilthead Sea Bream Fillets

Table 3 shows the proximate composition and fatty acid profile of the fillets of gilthead sea bream fed the three diets (CTRL, PAP, ALT). Dry matter, crude fat, protein, and ash contents did not differ significantly among diets. Regarding fatty acids (Table 3 and Table S5), the SFA proportion varied significantly (*p* < 0.001), being lowest in CTRL (23.5%) and highest in ALT (25.6%), mainly due to the contribution of lauric acid (C12:0). MUFA levels ranged from 42.3% (ALT) to 46.1% (CTRL), while the PUFA level was highest in PAP (21.8%). The total omega-3 fraction was greater in PAP (17.2%) and ALT (17.0%). The DHA content differed significantly (*p* < 0.001), with ALT showing the highest percentage (5.99%), and EPA percentages did not differ significantly. In addition, PUFA/SFA and MUFA/SFA ratios showed significant differences (*p* < 0.001). PAP exhibited the highest PUFA/SFA ratio (1.35), while CTRL had the highest MUFA/SFA ratio (1.96), indicating distinct lipid balance patterns among diets.

Table 4 provides the absolute amounts (g/100 g of fresh fillet) of key lipid components (MUFAs, PUFAs, C12:0, EPA, DHA), allowing a more practical interpretation of nutritional value for consumers. Absolute amounts have been calculated according to fat content in fish (Table 3) and the composition of the fatty acid profile. The content of EPA (g/100 g of fillet) varied significantly between 0.41 g/100 g (PAP) and 0.46 g/100 g (ALT). The amount of DHA present in g/100 g of fillet spanned from 0.41 g/100 g (CTRL) to 0.51 g/100 g (ALT). Therefore, fish fed the ALT diet had the highest levels of DHA and EPA (0.97 g/100 g). Lauric acid (C12:0) was also markedly higher in ALT (0.091 g/100 g). Indeed, medium-chain fatty acids (MCFAs), which are the sum of fatty acids between C8:00 and C12:0, were highest in the fillets of ALT fish (0.11 g/100 g). The total long-chain fatty acids (LCFAs), expressed as g per 100 g of fresh fillet, were higher in CTRL (8.65 g/100 g) and ALT (8.40 g/100 g).

**Figure 3:**
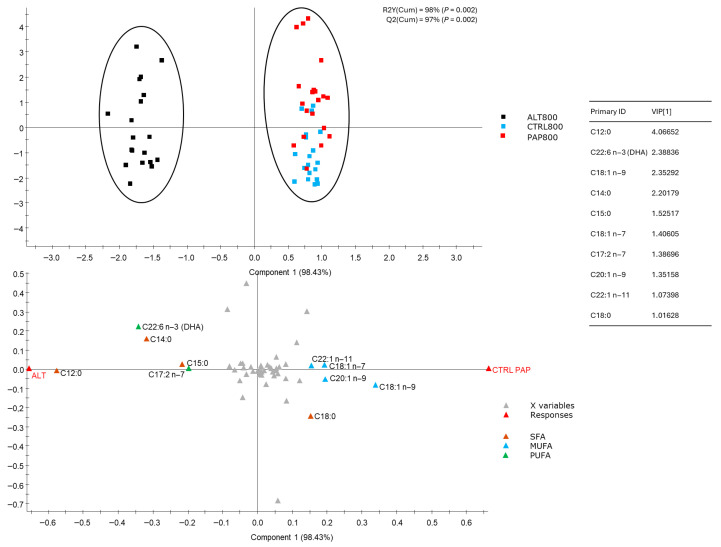
Two-dimensional representation of the distribution of *Sparus aurata* samples (n = 60) between the first two components of the partial least squares discriminant analysis (PLS-DA) model driving the separation of groups based on one component. The upper panel shows the classification of the samples by group. The lower panel (biplot) displays both the sample centroids and the explanatory variables (fatty acids), colored according to their class (SFAs, MUFAs, PUFAs). Additionally, the table associated with the model lists the fatty acids with the greatest contribution to group separation (components 1 and 2) (VIP > 1), indicating their relative importance in the projection.

These changes are reflected in the partial least squares discriminant analysis (PLS-DA) (Figure 3), which showed clear clustering of samples in two zones: ALT and CTRL/PAP grouped together [variance explained, R2Y(Cum): 98%; variance predicted, Q2Y(Cum): 97%]. Component 1 accounted for most of the variance explained (98.43%), driving the separation of groups (Figure 3 upper panel). Graphical representation of the contribution of each component to variance explained (R2Y) and predicted (Q2Y) in the PLS-DA model, driving the separation of groups based on the gilthead sea bream fatty acid profile, is shown in Figure S3: R^2^Y^2^ and Q^2^Y are both above 0.8, and their permutation *p*-value is 0.002, which is considered a highly robust and predictive model. The biplot (Figure 3 lower panel) displays sample centroids and fatty acids colored by class (SFAs, MUFAs, PUFAs). It highlights the variables exerting the most influence on group discrimination, defined by a VIP > 1. According to the VIP scores, ALT fillets were characterized by higher levels of C12:0 (lauric acid) and LCFAs, including C22:6n3 (DHA), C14:0, C17:2n-7, and C15:0, while CTRL and PAP fillets were associated with greater MUFA content, particularly oleic acid (C18:1n9), and also LCFAs such as C20:1n9, C18:1n7, C22:1n11, and C18:0.

### 3.4. Healthy Human Indices of Fish Fillets

Table 3 presents the fatty acid health indices that estimate health effects in humans of gilthead sea bream fed the three diets (CTRL, PAP, ALT). Significant differences were observed for most parameters, except the thrombogenic index (TI), hypercholesterolemic fatty acids (HFAs), and the n6/n3 ratio, which did not differ among diets. The LA/ALA ratio varied significantly (*p* < 0.001), ranging from 4.31 (CTRL) to 4.89 (PAP). The OA/SA also showed significant differences (*p* < 0.001), varying between 10.4 (PAP) and 11.4 (CTRL). The atherogenic index (AI) was lowest in CTRL (0.28) and highest in ALT (0.33). The hypocholesterolemic/hypercholesterolemic (HH) index varied significantly among the different diets (*p* < 0.001), being highest in CTRL (2.74), and lowest in ALT (2.52). Finally, the fish lipid quality (FLQ) index varied significantly, with ALT showing the highest values (11.4) and CTRL the lowest (9.68).

## 4. Discussion

The experimental diets (CTRL, PAP, and ALT) differed mainly in protein sources, and, to a minor degree, in lipid sources (Table S1). Within each feed type, composition varied by pellet size to match the nutritional needs of fish at different growth stages. For this reason, at early growing stages (2 mm feed), PAP and ALT formulations also contained fish protein sources (8%); such a diet lasted for 5.9% of the fishes’ lifetime (Figure S1) and afterwards was fully replaced with alternative protein sources. The protein content in the feed decreased as the growing stage progressed (from 50% to 44%) in all types of feed, whereas the fat content showed only minor adjustments (Table 1). No differences in the proximate compositions of the feeds were detected, whereas fatty acid compositions differed among feed formulations. These differences in fatty acid composition are linked not only to ingredient selection, but also to pellet size adjustments throughout development. Smaller pellets (early stages) tended to have slightly higher proportions of PUFAs, supporting rapid growth and metabolic demands, whereas larger pellets (later stages) showed a relative stabilization or slight dilution of these fractions. However, all diets included equal contents of FO as the main source of EPA and DHA, since no current alternatives fully replicate their benefits in aquaculture [31]. The PAP diet also contained salmon oil (7.2–3%), while the ALT diet used algae oil (less than 1%) as a sustainable DHA source. PAP was the diet with the highest content of marine oils, and, consistently, the one with the highest percentage of EPA and PUFAs (Table 2). Conversely, the ALT diet, having the lowest percentage of PUFAs, had the highest DHA levels, which is due to the DHA richness in algae oil (40% DHA and 16% EPA) [32]. It also had the highest SFA content, due to the C12:0 from insect meal made from the black soldier fly (*Hermetia illucens*), whose fat is about 50% lauric acid [33]. The CTRL diet contained the highest proportion of marine ingredients (fishmeal and fish oil), both of which contributing to the fat content of the feed. The experimental diets evaluated in the present study sustained growth performance in a manner comparable to the conventional control diet. Specifically, gilthead sea bream fed the CTRL, PAP, and ALT diets grew from approximately 13 g to 940–990 g over the production cycle, with indistinguishable specific growth rates (overall SGR 0.77–0.78) and feed conversion ratios (overall FCR 1.7–1.8), and without biologically relevant differences in weight gain or condition factors among treatments up to 2 years of age, while being supported by different physiological mechanisms [17,19]. Extrapolation of the present results should take into account the tank-based experimental design.

The fatty acid profiles of fillet composition revealed that CTRL-fed fish showed the highest MUFA levels, which is in accordance with the highest MUFA content in the diet. It is worth noting that C12:0 was present in fish fed the ALT diet at a level over 10 times higher than in the CTRL- and PAP-fed fish. Lauric acid was transferred from insect meal to feed and finally to fish fillets, with the highest levels of MCFAs seen in fish fed the ALT diet. From a human nutrition perspective, C12:0 levels should not be considered detrimental, as wild gilthead seabream fillets contain 0.21% of C12:0, which is an intermediate value among the three diets studied (Table 3) [34]. As for fat metabolism in fish, various studies have shown that MCFAs are able to diffuse more rapidly than LCFAs through the cellular membrane [35]. In this context, lauric acid has been reported to cross the mitochondrial membrane more readily than LCFAs, which typically require carnitine palmitoyltransferase-1 (CPT-1) for mitochondrial uptake [36]. For this reason, C12:0 is often considered a rapidly oxidized energy substrate. On this basis, it can be hypothesized that increased availability of lauric acid may reduce reliance on other lipid classes, including PUFAs, for energy metabolism. This putative mechanism could partly contribute to the higher EPA and DHA concentrations observed in ALT fillets, although no direct metabolic evidence is provided in the present study. The growth-promoting and metabolic effects of MCFA, particularly lauric acid, have been previously demonstrated in gilthead sea bream, where dietary supplementation with coconut-derived MCFAs enhanced intestinal architecture and nutrient absorption [37]. These findings are consistent with the hypothesis that C12:0 may act as a rapidly oxidized energy source, potentially influencing the retention of long-chain omega-3 fatty acids in muscle tissue. Similar effects have been reported in mammals, where lauric triglyceride supplementation in obese rats reduced lipogenesis and enhanced lipolysis and β-oxidation, along with increased mitochondrial biogenesis [38].

Multivariate analysis (PLS-DA) confirmed the influence of diet formulation on the overall fatty acid profile of feeds and fillets and pointed to the major contributors to differences. Virtual diet clustering showed clear separation among the CTRL, PAP, and ALT formulations, driven by MUFA and PUFA content in CTRL and PAP and higher SFA (notably C12:0) and DHA contents in ALT. Similarly, fillet profiles grouped ALT-fed fish apart from CTRL- and PAP-fed fish, indicating that the inclusion of insect meal and DHA-rich microalgae oil significantly modified the lipid composition. Freshwater fish are capable of synthesizing LC-PUFAs from linoleic acid (LA, 18:2n6) and linolenic acid (LNA, 18:3n3) through desaturation and elongation, while marine fish, such as *Sparus aurata*, lack this ability, mainly due to the absence of the Δ5-desaturase enzyme [39]. Therefore, the higher concentration of EPA and DHA in ALT-fed fish is unlikely to reflect increased PUFA synthesis and may instead be related to metabolic partitioning processes, potentially involving preferential oxidation of lauric acid and greater retention of ingested EPA and DHA for storage [35]. While some studies report no effect of insect meal, specifically from *Hermetia illucens*, on fish lipid profiles [40,41], studies using the alga *Pelvetia canaliculata* indicate that it improves the lipid profile of fish [26]. Our results confirm that combining insect meal with DHA-rich microalgae improves the lipid profile of gilthead seabream, increasing EPA and DHA levels. This ensures that alternative diets maintain or improve nutritional benefits without compromising health or product quality. A comprehensive understanding of ingredient profiles facilitates the accurate prediction of nutritional values.

Fish is a primary dietary sources of omega-3 fatty acids, and its high nutritional density is a principal justification for its recommended consumption. Considering the absolute amount of EPA and DHA in fillets, all diets provide levels that meet European Food Safety Authority (EFSA) nutritional recommendations for different population groups [42] (Table S6). Fish fed any of the experimental diets can be considered an excellent source of omega-3 fatty acids for all population groups. This is because a 100 g serving of fish from any of the diets analyzed exceeds the recommended intake for adults, children, pregnant women, and lactating women, as it provides approximately 800 to 970 mg of EPA+DHA. Furthermore, the lowest value observed (410 mg of DHA in fish fed the CTRL diet) is well above the threshold established for children, pregnant women, and lactating women.

In addition, European Union rules on nutritional claims are established in Regulation (EC) No 2006/1924 [7]. Nutritional claims provide information about the composition of the food. This regulation is the legal framework for food business operators to inform consumers through labelling regarding highly relevant food components, such as “high content of omega-3 fatty acids”. Health claims are established in Regulation (EC) No 2012/432 [43] and provide information about food effects on health; they may be used for advertising the beneficial health effects of foods, such as “DHA helps maintain normal brain function”. Many more claims were requested by companies; however, the European Commission authorized only 7 of the 54 health claims that had been requested on the potential health benefits of DHA (R(EU) 2012/432) and 3 more related to benefits for a specific population: pregnant women and/or children (R(EU) 440/2011) [44]. As for the EPA, there were 82 application requests, but only 3 were authorized. The unauthorized claims did not pass the evaluation for scientific evidence (R(EU) 2013/536), and it was pointed out that further research was needed on the other potential beneficial effects of EPA and DHA consumption. The authorized Health Claims for EPA and DHA (R2012/432) and nutritional claims under European Union regulation 2006/1924 are presented in Table S6. Based on the fatty acid composition of fillets, and the amount of DHA and EPA in 100 g of fillet fish, for such a serving size, we can apply Health Claim 1, Health Claim 2, and Health Claim 6 to all diets. In addition, all experimental diets meet the conditions for the authorized nutritional claims under EU regulations. Importantly, the use of alternative feeds does not compromise these properties, and levels of essential fatty acids remain within ranges associated with health benefits. This demonstrates that fish produced with the tested sustainable diets can deliver the same nutritional advantages as those fed traditional formulations, ensuring compliance with labeling requirements while supporting aquaculture sustainability.

The healthy human indices calculated for gilthead sea bream specimens fed with the three experimental diets provide valuable insights into the nutritional quality of the fish lipid fraction. The PUFAs/SFAs and n6/-3 ratios obtained are comparable to those reported in previous studies evaluating the proximate composition and fatty acid profile of gilthead sea bream fed diets supplemented with *Pelvetia canaliculata* (PUFAs/SFAs = 1.43; n6 = 1.22) [26]. In our case, the closest values were observed in the PAP diet (1.35).

The AI, which reflects the ratio of SFAs to unsaturated fatty acids (UFAs), ranged from 0.28 to 0.33 in our study. These values are within the expected range (0.21–1.14) described in the food literature for fish [45]. Other studies in gilthead sea bream reported similar values (0.21–0.29) [46]. The observed values are considered low, and a low-AI diet is expected to reduce total cholesterol and LDL-C levels in human blood plasma [47]. Regarding TI, lower values indicate a reduced tendency to form thrombi, which is beneficial for cardiovascular health [45]. Fish fillets showed a TI of 0.28, consistent with the reference interval reported for fish (0.139–0.56) and markedly lower than other foods that are considered healthy, such as rabbit meat, which has values of 0.83–1.12 [48].

A relatively high HH with low AI and TI is expected to contribute to a decreased incidence of coronary heart disease [49]. The HH index in our gilthead sea bream fillets ranged between 2.52 and 2.74, which is close to other values reported for marine fish species from the Atlantic Ocean (0.87–2.46) [50]. These values are relatively high compared to reference values for other foods such as cow’s milk (0.34–0.75) [51]. A high FLQ value indicates a high proportion of long-chain omega-3 fatty acids (EPA and DHA) in the total fatty acid profile, which contributes to a better nutritional quality of the lipid source [52]. In our study, FLQ values (9.68–11.4) were substantially higher than those reported for gilthead sea bream farmed in the Adriatic Sea [53], where the average was 3.06. This difference likely results from feed formulation, as commercial diets often include higher proportions of vegetable oils [54] than those in the present study. The evaluated experimental diets incorporated fish oil and microalgae as sources of EPA and DHA, in addition to the vegetable oil (rapeseed oil). Thus, our FLQ values represent a significant improvement compared to conventional diets and confirm that sustainable feed alternatives can maintain lipid quality at levels beneficial for human nutrition.

Taken together, the preservation of lipid nutritional quality and compliance with health claim requirements highlight the importance of also considering consumer perception when evaluating alternative aquafeed formulations. Beyond zootechnical performance and fillet nutritional attributes, the adoption of alternative protein and lipid sources in aquafeeds must ultimately ensure product quality and consumer acceptance. In gilthead sea bream, previous studies have shown that replacing fish oil with microalgae-derived oils does not negatively affect the sensory attributes of the fillet [55]. Similarly, diets including defatted black soldier fly meal at inclusion levels up to 15% did not result in off flavors or have adverse effects on overall sensory quality, despite minor differences in specific descriptive attributes [56].

Despite the insights provided by the study, several limitations must be acknowledged. The hypothetical effect on C12:0 on fatty acid metabolism remains to be confirmed. Further studies incorporating direct metabolic measurements would be beneficial to provide a more comprehensive understanding.

## 5. Conclusions

The findings from this long-term trial study highlight the feasibility of using sustainable feed alternatives in gilthead sea bream without compromising its fatty acid profile or its potential health benefits for human consumption. The formulations developed with alternative protein sources, mainly replacing fishmeal-based ingredients, are able to maintain polyunsaturated fatty acid (EPA and DHA) levels necessary to ensure fish growth. Reasonable serving sizes of fish fillets can cover the needs and recommendations for EPA and DHA for all consumer groups. Fish fed the ALT diet have the highest levels of EPA and DHA, even though they ingested the lowest amount of omega-3. The intake of algae oil rich in DHA, together with the C12:0 supplied from insect meal, may contribute to metabolic conditions that favor omega-3 accumulation in fish muscle, although the underlying mechanisms require further investigation. The overall health lipid indices of the fish fillets (e.g., PUFAs/SFAs, n6/n3, AI, TI) remain within favorable nutritional ranges. Some variations, particularly in the PUFAs/SFAs and n6/n3 ratios, reflect the influence of dietary formulation on the lipid quality of the fillets. These results underscore the importance of sustainable feeding strategies in aquaculture, promoting marine resource conservation while maintaining the nutritional value and health-promoting properties of farmed fish. From an applied aquaculture perspective, these findings support feed formulation strategies that combine alternative protein sources with targeted lipid inputs as a viable approach to reduce reliance on fishmeal and fish oil. The consistency of lipid quality indicators across dietary treatments suggests that such formulations could be progressively integrated into commercial feeding programs without compromising production efficiency. Taken together, these outcomes provide practical guidance for the development of sustainable aquafeeds capable of maintaining product quality under farming-relevant conditions.

## Figures and Tables

**Table 1:** Proximal composition of feeds with different formulations and particle sizes. Crude fat, protein, crude fiber, ash, and organic matter are expressed as percentages of dry weight, while dry matter is expressed as a percentage of fresh weight. Data were analyzed using the non-parametric Kruskal–Wallis test (one-way ANOVA on ranks). When significant differences were observed, multiple post hoc comparisons were performed using a stepwise step-down procedure to identify specific group differences. Statistical analyses were conducted with IBM SPSS Statistics, and significance was set at *p* < 0.05. The statistics were calculated using technical replicates (n = 36).

		Crude Fat	Protein	Crude Fiber	Ash	Organic Matter	Dry Matter
		Kruskal–Wallis					
Fish feed formulation	Particle size (mm)	**	**	**	***	***	**
CTRL	2.0	17.7 ± 0.26 ^abc^	50.3 ± 0.31 ^cd^	1.41 ± 0.02 ^a^	7.83 ± 0.01 ^i^	87.5 ± 0.01 ^e^	95.2 ± 0.05 ^cd^
	3.0	18.8 ± 0.80 ^cd^	47.8 ± 0.10 ^bcd^	1.85 ± 0.19 ^abcd^	7.97 ± 0.02 ^j^	88.2 ± 0.003 ^f^	96.4 ± 0.05 ^e^
	4.5	17.0 ± 0.16 ^a^	48.6 ± 0.89 ^bcd^	1.85 ± 0.04 ^abcd^	7.80 ± 0.04 ^h^	86.6 ± 0.04 ^c^	95.0 ± 0.09 ^abc^
	6.0	17.0 ± 0.09 ^a^	44.5 ± 0.40 ^abc^	1.66 ± 0.04 ^ab^	8.59± 0.03 ^k^	85.5 ± 0.17 ^a^	94.1 ± 0.05 ^ab^
PAP	2.0	18.3 ± 0.04 ^bcd^	51.0 ± 0.18 ^d^	1.43 ± 0.10 ^a^	7.12 ± 0.05 ^f^	88.7 ± 0.06 ^g^	94.4 ± 0.05 ^abc^
	3.0	19.0 ± 2.75 ^cd^	48.2 ± 0.51 ^bcd^	1.70 ± 0.14 ^ab^	6.27 ± 0.05 ^b^	89.0 ± 0.04 ^h^	95.1 ± 0.05 ^bcd^
	4.5	16.3 ± 0.55 ^a^	48.5 ± 0.84 ^bcd^	1.99 ± 0.04 ^bcde^	5.88 ± 0.02 ^a^	86.4 ± 0.02 ^b^	94.3 ± 0.09 ^abc^
	6.0	17.2 ± 0.14 ^ab^	43.2± 0.26 ^a^	1.83 ± 0.08 ^abcd^	6.47 ± 0.03 ^c^	86.9 ± 0.07 ^d^	93.4 ± 0.05 ^a^
ALT	2.0	18.1 ± 0.66 ^bcd^	48.5 ± 0.21 ^bcd^	1.73 ± 0.06 ^abc^	7.36 ± 0.05 ^g^	86.2 ± 0.05 ^a^	95.9 ± 0.04 ^d^
	3.0	19.6 ± 0.27 ^d^	47.9 ± 0.04 ^bcd^	2.05 ± 0.06 ^cde^	6.64 ± 0.05 ^d^	88.5 ± 0.07 ^fg^	95.3 ± 0.14 ^cd^
	4.5	16.6 ± 0.38 ^a^	48.2 ± 2.84 ^bcd^	2.51 ± 0.02 ^e^	6.14 ± 0.02 ^b^	88.4 ± 0.02 ^fg^	94.3 ± 0.03 ^abc^
	6.0	17.9 ± 0.15 ^bcd^	43.3 ± 0.34 ^ab^	2.01 ± 0.13 ^de^	6.82 ± 0.01 ^e^	84.9 ± 0.60 ^a^	92.8 ± 1.70 ^abc^

Least square means within the same column that are denoted by different letters differ significantly. ** *p* < 0.01; *** *p* < 0.001. ± standard error. CTRL: fishmeal; PAP: processed animal protein–based diet; ALT: Alternative protein.

**Table 2:** Profile of essential and major fatty acids (% of total fatty acids) in feed formulations and particle size (*n* = 12). Data were analyzed using the non-parametric Kruskal–Wallis test (one-way ANOVA on ranks). When significant differences were observed, multiple post hoc comparisons were performed using a stepwise step-down procedure to identify specific group differences. Statistical analyses were conducted with IBM SPSS Statistics, and significance was set at *p* < 0.05. The statistics were calculated using technical replicates (n = 36).

		SFAs	MUFAs	PUFAs	EPA	DHA	Omega-3 Fatty Acids
		Kruskal–Wallis					
Fish feed formulation	Particle size (mm)	***	***	**	***	***	**
CTRL	2.0	22.5 ± 0.08 ^b^	45.2 ± 0.30 ^f^	32.3 ± 0.05 ^a^	6.20 ± 0.09 ^abc^	5.29 ± 0.09 ^h^	16.5 ± 0.23 ^abc^
	3.0	22.8 ± 0.10 ^c^	46.9 ± 0.55 ^g^	30.3 ± 0.31 ^a^	5.40 ± 0.22 ^a^	3.96 ± 0.08 ^d^	14.3 ± 0.47 ^a^
	4.5	23.0 ± 0.07 ^cd^	42.9 ± 0.11 ^cde^	34.1 ± 0.11 ^c^	6.97 ± 0.07 ^cde^	4.37 ± 0.01 ^de^	18.1 ± 0.01 ^cd^
	6.0	24.1 ± 0.12 ^e^	43.1 ± 0.15 ^de^	32.8 ± 0.11 ^bc^	7.86 ± 0.16 ^ef^	2.75 ± 0.02 ^a^	15.9 ± 0.25 ^ab^
PAP	2.0	26.4 ± 0.15 ^g^	39.9 ± 0.22 ^a^	33.8 ± 0.06 ^ab^	6.80 ± 0.05 ^bcd^	5.78 ± 0.03 ^i^	17.1 ± 0.09 ^bcd^
	3.0	21.9 ± 0.05 ^a^	43.9 ± 0.26 ^e^	34.2 ± 0.16 ^bc^	6.83 ± 0.11 ^cd^	5.01 ± 0.02 ^gh^	17.4 ± 0.22 ^bcd^
	4.5	23.3 ± 0.12 ^bcde^	42.7 ± 0.47 ^bcde^	34.0 ± 0.39 ^bc^	6.87 ± 0.12 ^cd^	4.64 ± 0.08 ^efg^	18.1 ± 0.31 ^d^
	6.0	24.7 ± 0.16 ^f^	42.1 ± 0.23 ^bcd^	33.2 ± 0.23 ^bc^	8.27 ± 0.09 ^f^	2.97 ± 0.02 ^b^	17.0 ± 0.16 ^bcd^
ALT	2.0	25.9 ± 0.05 ^g^	41.3 ± 0.29 ^abc^	32.8 ± 0.17 ^a^	6.15 ± 0.10 ^ab^	5.94 ± 0.07 ^i^	16.9 ± 0.23 ^bcd^
	3.0	23.7 ± 0.19 ^de^	43.4 ± 0.10 ^de^	32.9 ± 0.29 ^abc^	6.15 ± 0.04 ^ab^	4.97 ± 0.04 ^fgh^	16.7 ± 0.29 ^abcd^
	4.5	26.1 ± 0.10 ^g^	41.4 ± 0.49 ^abcd^	32.4 ± 0.12 ^a^	6.94 ± 0.25 ^cd^	4.53 ± 0.16 ^def^	17.2 ± 0.57 ^bcd^
	6.0	27.4 ± 0.23 ^h^	40.8 ± 0.49 ^ab^	31.8 ± 0.19 ^a^	7.87 ± 0.12 ^def^	3.15 ± 0.03 ^c^	16.1 ± 0.18 ^ab^

SFAs (saturated fatty acids); MUFAs (monounsaturated fatty acids); PUFAs (polyunsaturated fatty acids); EPA (eicosapentaenoic acid); DHA (docosahexaenoic acid); least square means within the same column that are denoted by different letters differ significantly. ** *p* < 0.01; *** *p* < 0.001. ± standard error. CTRL: fishmeal; PAP: processed animal protein–based diet; ALT: alternative protein.

**Table 3:** Proximate composition (% in wet weight), profile of essential and major fatty acids (% of total fatty acids), and health indices of *Sparus aurata* samples (*n* = 60) corresponding to different fish feed formulations (FM, PAP, and ALT). Data were analyzed using the non-parametric Kruskal–Wallis test (one-way ANOVA on ranks). When significant differences were observed, multiple post hoc comparisons were performed using a stepwise step-down procedure to identify specific group differences. Statistical analyses were conducted with IBM SPSS Statistics, and significance was set at *p* < 0.05. The statistics were calculated using biological replicates.

	Kruskal–Wallis	CTRL	PAP	ALT
*Proximate composition*				
Crude fat	NS	8.67 ± 0.99	7.66 ± 0.7	8.51 ± 0.9
Protein	NS	17.08 ± 0.8	18.52 ± 0.6	17.81 ± 0.9
Ash	NS	1.42 ± 0.15	1.28 ± 0.16	1.28 ± 0.27
Dry matter	NS	28.44 ± 1.15	28.20 ± 1.97	27.53 ± 3.03
*Profile of essential and major fatty acids (% total fatty acid profile)*				
SFAs	***	23.5 ± 0.136 ^a^	24.4 ± 0.215 ^b^	25.6 ± 0.222 ^c^
MUFAs	***	46.1 ± 0.194 ^b^	42.8 ± 0.328 ^a^	42.3 ± 0.294 ^a^
PUFAs	***	20.7 ± 0.118 ^a^	21.8 ± 0.339 ^b^	20.7 ± 0.109 ^a^
Omega-3 fatty acids	**	15.6 ± 0.244 ^a^	17.2 ± 0.540 ^b^	17.0 ± 0.403 ^b^
EPA	NS	4.97 ± 0.192	5.37 ± 0.320	5.35 ± 0.300
DHA	***	4.71 ± 0.293 ^a^	5.56 ± 0.356 ^b^	5.99 ± 0.430 ^b^
C12:0	***	0.05 ± 0.032 ^a^	0.07 ± 0.046 ^b^	1.10 ± 0.107 ^c^
SCFAs	NA	NA	NA	NA
MCFAs	***	0.18 ± 0.129 ^a^	0.26 ± 0.190 ^b^	1.29 ± 0.115 ^c^
LCFAs	***	99.8 ± 0.005 ^c^	99.7 ± 0.009 ^b^	98.7 ± 0.013 ^a^
*Fatty acid ratios*				
PUFAs/SFAs	***	1.29 ± 0.047 ^b^	1.35 ± 0.101 ^b^	1.26 ± 0.051 ^a^
MUFAs/SFAs	***	1.96 ± 0.068 ^c^	1.76 ± 0.095 ^b^	1.66 ± 0.095 ^a^
*Health indices*				
n6/n3	NS	0.85 ± 0.054	0.81 ± 0.107	0.81 ± 0.083
LA/ALA	***	4.31 ± 0.065 ^a^	4.89 ± 0.208 ^b^	4.86 ± 0.189 ^b^
OA/SA	**	11.4 ± 0.347 ^b^	10.4 ± 0.354 ^a^	11.3 ± 0.425 ^b^
AI	***	0.28 ± 0.025 ^a^	0.30 ± 0.038 ^b^	0.33 ± 0.040 ^c^
TI	NS	0.28 ± 0.022 ^b^	0.28 ± 0.050 ^ab^	0.28 ± 0.043 ^a^
HFAs	NS	22.1 ± 0.148 ^a^	22.8 ± 0.231 ^ab^	22.7 ± 0.228 ^b^
HH	***	2.74 ± 0.080 ^b^	2.55 ± 0.110 ^a^	2.52 ± 0.103 ^a^
FLQ	***	9.68 ± 0.325 ^a^	10.9 ± 0.451 ^b^	11.4 ± 0.495 ^b^

CTRL: fishmeal; PAP: processed animal protein–based diet; ALT: alternative protein; least square means within the same column that are denoted by different letters differ significantly. ** *p* < 0.01; and *** *p* < 0.001. NS: not significant. NA: not available. ± standard error. SFAs (saturated fatty acids); MUFAs (monounsaturated fatty acids); PUFAs (polyunsaturated fatty acids); EPA (eicosapentaenoic acid); DHA (docosahexaenoic acid); SCFAs (short-chain fatty acids (sum of <C6:0)); MCFAs (medium-chain fatty acids (sum of C8:0–C12:0)); LCFAs (long-chain fatty acids (sum of >C14:0)); PUFAs/SFAs (polyunsaturated fatty acids/saturated fatty acids); MUFAs/SFAs (monounsaturated fatty acids/saturated fatty acids); n-6/n-3 (omega-6/omega-3); LA/ALA (linoleic acid/α-linoleic acid); OA/SA (oleic acid/stearic acid); IA (atherogenicity index); IT (thrombogenicity index); HFA (hypercholesterolemic fatty acids); HH (hypocholesterolemic/hypercholesterolemic ratio); FLQ (fish lipid quality).

**Table 4:** MUFAs, PUFAs, C12:0, EPA, and DHA content expressed as g/100 g fresh fillet of *Sparus aurata* samples (*n* = 60) corresponding to different feed formulations (FM, PAP, and ALT). Data were analyzed using the non-parametric Kruskal–Wallis test (one-way ANOVA on ranks). When significant differences were observed, multiple post hoc comparisons were performed using a stepwise step-down procedure to identify specific group differences. Statistical analyses were conducted with IBM SPSS Statistics, and significance was set at *p* < 0.05. The statistics were calculated using biological replicates.

	Kruskal–Wallis	CTRL	PAP	ALT
MUFAs	***	4.00 ± 0.017 ^c^	3.28 ± 0.025 ^a^	3.60 ± 0.025 ^b^
PUFAs	***	2.63 ± 0.010 ^b^	2.51 ± 0.026 ^a^	2.73 ± 0.009 ^b^
EPA	***	0.43 ± 0.017 ^b^	0.41 ± 0.025 ^a^	0.46 ± 0.026 ^b^
DHA	***	0.41 ± 0.025 ^a^	0.43 ± 0.027 ^a^	0.51 ± 0.037 ^b^
EPA+DHA	***	0.84 ± 0.042 ^a^	0.84 ± 0.052 ^a^	0.97 ± 0.063 ^b^
C12:0	***	0.004 ± 0.003 ^a^	0.005 ± 0.004 ^a^	0.091 ± 0.009 ^b^
SCFAs	NA	NA	NA	NA
MCFAs	***	0.02 ± 0.011 ^a^	0.02 ± 0.015 ^a^	0.11 ± 0.010 ^b^
LCFAs	***	8.65 ± 0.000 ^c^	7.64 ± 0.001 ^a^	8.40 ± 0.001 ^b^

Least square means within the same column that are denoted by different letters differ significantly *** *p* < 0.001 Anot available. ± standard error. MUFAs (monounsaturated fatty acids); PUFAs (polyunsaturated fatty acids); EPA (eicosapentaenoic acid); DHA (docosahexaenoic acid); SCFAs (short-chain fatty acids (sum of <C6:0)); MCFAs (medium-chain fatty acids (sum of C8:0-C12:0)); LCFAs (long-chain fatty acids (sum of >C14:0)).

## Data Availability

The original contributions presented in this study are included in the article/Supplementary Materials. Further inquiries can be directed to the corresponding author.

## Supplementary Materials

The following supporting information can be downloaded at: https://www.mdpi.com/article/10.3390/foods15101762/s1, Figure S1: Percentage intake of feed of size 2, 3, 4.5, and 6 mm for different diets (CTRL_VF800, PAP_VF800, ALT_VF800); Figure S2: Graphical representation of the contribution of each of the two principal components to variance explained (R2Y, grey) and predicted (Q2Y, black) in the PLS-DA model, driving the separation of groups two components. Validation of the model by random permutations (right); Figure S3: Graphical representation of the contribution of each of the two principal components to variance explained (R2Y, grey) and predicted (Q2Y, black) in the PLS-DA model, driving the separation of groups based on fish size (component 1) and fish feed formulation (component 2). Validation of the model by random permutations (right); Table S1: Ingredients of experimental diets with a pellet size of 2, 3, 4.5, and 6 mm. Values of pellet size of 2 mm, 3 mm, 4.5 mm, and 6 mm are in bold, red, and regular font, respectively, if variations are present. Otherwise, a unique value is shown; Table S2: Gas chromatograph operating parameters; Table S3: Description and calculation of fatty acid ratios and human health indexes; Table S4: Concentrations of the analyzed FAMEs (% of total fatty acids) in different fish feed formulations (CTRL, PAP, ALT) (n = 12); Table S5: Concentrations of the analyzed FAMEs (% of total fatty acids) in *Sparus aurata* fed different fish feed formulations (CTRL, PAP, ALT) (n = 60); Table S6: Nutritional recommendations, Health Claims, and Nutritional Claims established in Regulation (EU) Nº 432/2012 and Regulation (EU) Nº 1924/2006.
