# Supplementary material for: Evaluating Sustainable Feed Alternatives in Sparus aurata: How Alternative Proteins and Oils Maintain EPA+DHA Content and Improve Human Health Lipid Indices
Source: Foods. 2026 May 16;15(10):1762. doi: 10.3390/foods15101762 (PMC13206125; doi:10.3390/foods15101762)
Supplement: Supplementary file 1 [file foods-15-01762-s001.zip › Figures Supplementary_FOODS.pdf]

**Growth stage: 800 g**

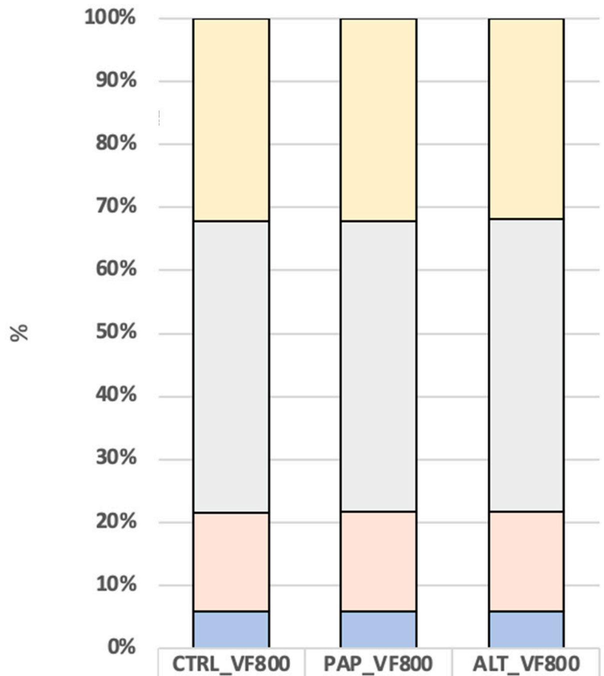


| | CTRL_VF800 | PAP_VF800 | ALT_VF800 |
|---|---|---|---|
| 6 mm (%) | 32,1 | 32,2 | 31,9 |
| 4.5 mm (%) | 46,4 | 46,2 | 46,6 |
| 3 mm (%) | 15,6 | 15,8 | 15,7 |
| 2 mm (%) | 5,9 | 5,9 | 5,9 |

**Figure S1**. Percentage intake of feed of size 2, 3, 4,5 and 6 mm for different diets (CTRL_VF800, PAP_VF800, ALT_VFf800)

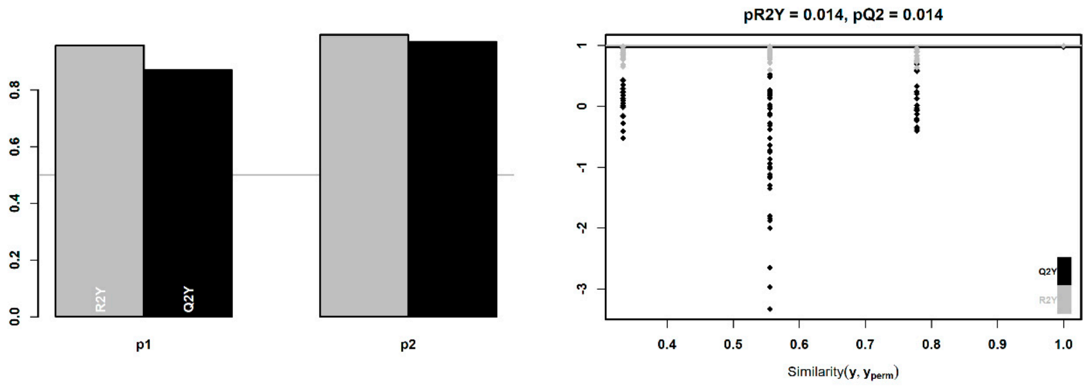


**Figure S2**. Graphical representation of the contribution of each of the two principal components to variance explained (R2Y, grey) and predicted (Q2Y, black) in PLS-DA model, driving the separation of groups two components. Validation of the model by random permutations (right).

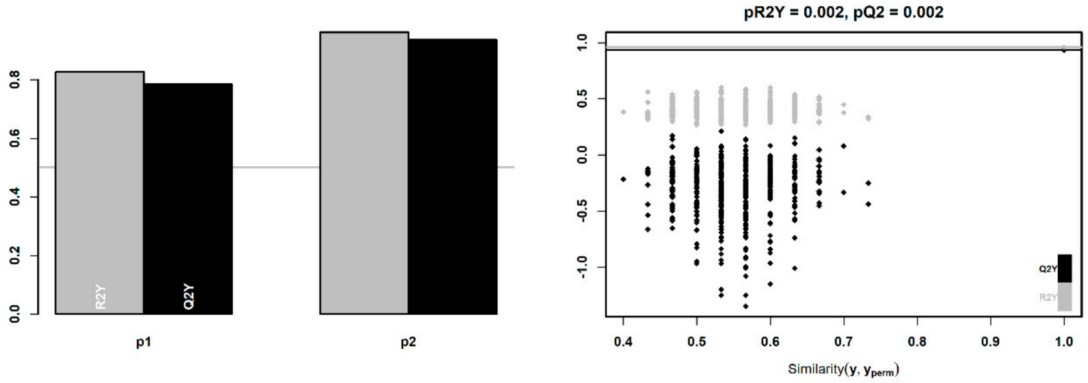


**Figure S3**. Graphical representation of the contribution of each of the two principal components to variance explained (R2Y, grey) and predicted (Q2Y, black) in PLS-DA model, driving the separation of groups based on fish size (component 1) and fish feed formulation (component 2). Validation of the model by random permutations (right).
