# Supplementary material for: Evaluating Sustainable Feed Alternatives in Sparus aurata: How Alternative Proteins and Oils Maintain EPA+DHA Content and Improve Human Health Lipid Indices
Source: Foods. 2026 May 16;15(10):1762. doi: 10.3390/foods15101762 (PMC13206125; doi:10.3390/foods15101762)
Supplement: Supplementary file 1 [file foods-15-01762-s001.zip › Tables Supplementary_R2.pdf]

**Table S1.** Ingredients of experimental diets with a pellet size of 2, 3, 4.5, and 6 mm. Values of pellet size of 2 mm, 3, 4.5 mm, and 6 mm are in bold, red, and regular font, respectively, if variations are present. Otherwise, a unique value is shown.

| | CTRL | PAP | ALT |
|---|---|---|---|
| **Ingredient (%)** | | | |
| Fishmeal Super Prime | **17** - 5 - 5 | 0 | 0 |
| Fishmeal 60 | **5** - 10 - 10 | **5** - 0 - 0 | **5** - 0 - 0 |
| Fish protein hydrolysate | **3** - 0 - 0 | **3** - 0 - 0 | **3** - 0 - 0 |
| Poultry meal | 12 | **25** - 17 - 17 | 12 |
| Porcine blood meal | 5 | 8 | 5 |
| Feathermeal hydrolysate | **5** - 7.25 - 7.25 | **5** - 9.75 - 9.5 | **5** - 7.25 - 7.25 |
| Insect meal (PROTE - IN HP55) | 0 | 0 | **12** – 8.5 - 8 |
| Aminopro NT70 | 0 | 0 | **6** - 5 - 5 |
| Corn gluten meal | **7** - 8 - 8 | **7** - 8 - 8 | **7** - 8 - 8 |
| Soybean meal 44 | **9** - 12 - 12 | **9** - 12 - 12 | **9** - 12 - 12 |
| Sunflower meal 40 | **4.5** - 7.5 - 7.5 | **4.5** - 7.5 - 7.5 | **4.5** - 7.5 - 7.5 |
| Wheat meal | **11.28** - 9.58 - 8.58 | **11.73** - 11.58 - 10.88 | **8.88** - 8.88 - 8.38 |
| Whole peas | 5 | 5 | 5 |
| Pea starch (raw) | 2 | 2 | 2 |
| Vitamin and mineral premix[1] | 1 | 1 | 1 |
| Vitamin C35 | **0** - 0 - 0.05 | **0** - 0 - 0.05 | **0** - 0 - 0.05 |
| Vitamin E50 | **0.1** - 0.1 - 0.15 | **0.1** - 0.1 - 0.15 | **0.1** - 0.1 - 0.15 |
| Antioxidant | 0.2 | 0.2 | 0.2 |
| Sodium propionate | 0.1 | 0.1 | 0.1 |
| MAP (Monoamonium phosphate) | **0.5** - 0.8 - 0.8 | **0.75** - 1.9 - 2.15 | **1.55** - 2.1 - 2.3 |
| Nucleotides (Nucleoforce AQUA) | **0** - 0 - 0.2 | **0** - 0 - 0.2 | **0** - 0 - 0.2 |
| l - Lysine HCl 99% | **0** - 0.4 - 0.4 | **0** - 0.6 - 0.6 | **0.3** - 0.7 - 0.7 |
| DL - Methionine | 0 | **0** - 0.1 - 0.1 | **0** - 0.1 - 0.1 |
| L - Taurine | **0** - 0 - 0.4 | **0** - 0 - 0.4 | **0** - 0 - 0.4 |
| Yttrium oxide | 0.02 | 0.02 | 0.02 |
| Rapeseed lecithin liquid | **0** - 0 - 0.5 | **0** - 0 - 0.5 | **0** - 0 - 0.5 |
| Fish oil | **5.4** - 6.65 - 6.65 | **5.4** - 6.65 - 6.65 | **5.4** - 6.65 - 6.65 |
| Salmon oil | 0 | **7.2** - 3 - 3 | 0 |
| Algae oil (Veramaris) | 0 | 0 | **0.9** - 0.4 - 0.4 |
| Rapeseed oil | **6.9** - 7.4 - 7.2 | **0** - 5.5 - 5 | **6.05** - 7.5 - 7.1 |

[1]Vitamin and mineral premix: Vitamins (IU or mg/kg diet): DL - alpha tocopherol acetate, 565 mg; sodium menadione bisulphate, 23.5 mg; retinyl acetate, 21,750 IU; DL - cholecalciferol, 4,640 IU; thiamine, 28.3 mg; riboflavin, 28.6 mg; pyridoxine, 22 mg; cyanocobalamin, 0.1 mg; nicotinic acid, 205 mg; folic acid, 14 mg; ascorbic acid, 935 mg; inositol, 465 mg; biotin, 2.9 mg; calcium pantothenate, 95.5 mg; choline chloride, 1070 mg; betaine, 465 mg. Mineral (g or mg/kg diet): Na, 3 g; Mg, 1.4 g; K, 5.9 g; Cu, 15.6 mg; Fe, 240 mg; I, 0.95 mg; Mn, 19.9 mg; Se, 0.5 mg; Zn, 48 mg.

**Table S2.** Gas Chromatograph operating parameters

| **FAMEs determination by GC-FID (Shimadzu 2010)** | |
|---|---|
| Injector (ratio) | Split (1:20) |
| Injector temperature | 250ºC |
| Injection volume | 1 µL |
| Capillary column | CP-Sil 88 (100 m * 0,25 mm * 0,20 µm, Agilent Technologies) |
| Carrier gas | Helium |
| Carrier gas flow | 1.05 L/min |
| Column oven temperature program | Stage 1: maintain at 45ºC for 4 min |
| | Stage 2: ramp to 175ºC for 10 min |
| | Stage 3: maintain at 175ºC for 27 min |
| | Stage 4: ramp to 215ºC for 10 min |
| | Stage 5: maintain at 215ºC for 35 min |
| Total program time | 86 min |
| Detector | FID |
| FID temperature | 260ºC |
| Flame gases and flow | Hydrogen 30mL/min and Synthetic Air 300mL/min |
| Make-up gas and flow | Nitrogen 45 ml/min |
| Compound identification | Retention time of certified standard mixtures: Certificate of Analysis (Supelco 37 Component FAME MIX); Bacterial Acid Methyl Esters CP Mixture (Lipids and Biochemicals); Certificate of Composition PUFA-1, Marine Source (Lipids and Biochemicals); Certificate of Composition PUFA-3, Marine Source (Lipids and Biochemicals) |

**Table S3.** Description and calculation of fatty acid ratios and human health indexes

| Code | Description | Mathematical equation |
|---|---|---|
| PUFA/SFA | Polyunsaturated Fatty Acids/Saturated Fatty Acids | ΣPUFA / ΣSFA |
| MUFA/SFA | Monounsaturated Fatty Acid/Saturated Fatty Acids | ΣMUFA / ΣSFA |
| n6/n3 | Omega 6/Omega 3 | (C18:2n6t +C18:2n6 + C18:3n6 + C20:2n6 + C20:3n6 + C20:4n6 + C22-2n6 + C24:2n6 + C22:5n6) / (C18:3n3 + C18:4n3 + C20:3n3 + C20:4n3 + C20:5n3 + C21:5n3 + C22:5n3 + C22:6n3) |
| LA/ALA | Lionoleic Acid/α-Linoleic Acid | C18:2n6 / C18:3n3 |
| OA/SA | Oleic acid/Stearic acid | C18:1n9 / C18:0 |
| IA | Atherogenicity index | (C12:0 + 4 x C14:0 + C16:0) / (MUFA + n3 + n6) |
| IT | Thrombogenicity index | (C14:0 + C16:0 + C18:0) / (0.5 x MUFA + 3 x n3 + 0.5 x n6 + n3 / n6) |
| HFA | Hypercholesterolemic index | ΣC12:0 + C14:0 + C16:0 |
| HH | Hypocholesterolemic/Hypercholesterolemic ratio | (C18:1n9 + C18:1n7 + C18:2n6 + C18:3n6 + C18:3n3 + C20:3n6 + C20:4n6 + C20:5n3 + C22:5n3 + C22:6n3) / (C12:0 + C14:0 + C16:0) |
| FLQ | Fish Lipid Quality | 100 * (DHA+EPA) / TFA |

*UFA: MUFA + PUFA; TFA: total fatty acids

**Table S4.** Concentrations of the analyzed FAMEs (% of total fatty acids) in different fish feed formulations (CTRL, PAP, ALT) (n = 12).

| | Code | Min-Max | Median | Average * | p-value |
|---|---|---|---|---|---|
| C4:0 | CTRL2 | 0.00-0.09 | 0.00 | 0.03 | 0.071 |
| | PAP2 | 0.00-0.00 | 0.00 | 0.00 | |
| | ALT2 | 0.00-0.00 | 0.00 | 0.00 | |
| | CTRL3 | 0.00-0.00 | 0.00 | 0.00 | |
| | PAP3 | 0.00-0.00 | 0.00 | 0.00 | |
| | ALT3 | 0.00-0.00 | 0.00 | 0.00 | |
| | CTRL4.5 | 0.00-0.11 | 0.10 | 0.07 | |
| | PAP4.5 | 0.00-0.00 | 0.00 | 0.00 | |
| | ALT4.5 | 0.00-0.00 | 0.00 | 0.00 | |
| | CTRL6 | 0.00-0.00 | 0.00 | 0.00 | |
| | PAP6 | 0.00-0.00 | 0.00 | 0.00 | |
| | ALT6 | 0.00-0.00 | 0.00 | 0.00 | |
| C10:0 | CTRL2 | 0.00-0.00 | 0.00 | 0.00 a | <0.001 |
| | PAP2 | 0.00-0.00 | 0.00 | 0.00 a | |
| | ALT2 | 0.00-0.07 | 0.06 | 0.04 ab | |
| | CTRL3 | 0.00-0.00 | 0.00 | 0.00 a | |
| | PAP3 | 0.00-0.00 | 0.00 | 0.00 a | |
| | ALT3 | 0.00-0.06 | 0.06 | 0.04 abc | |
| | CTRL4.5 | 0.10-0.11 | 0.11 | 0.11 bc | |
| | PAP4.5 | 0.10-0.12 | 0.11 | 0.11 b | |
| | ALT4.5 | 0.10-0.11 | 0.10 | 0.11 bc | |
| | CTRL6 | 0.00-0.00 | 0.00 | 0.00 a | |
| | PAP6 | 0.00-0.00 | 0.00 | 0.00 a | |
| | ALT6 | 0.08-0.11 | 0.10 | 0.10 bc | |
| C12:0 | CTRL2 | 0.06-0.09 | 0.07 | 0.07 abc | <0.001 |
| | PAP2 | 0.06-0.06 | 0.06 | 0.06 a | |
| | ALT2 | 3.10-3.52 | 3.29 | 3.30 d | |
| | CTRL3 | 0.06-0.14 | 0.06 | 0.09 abc | |
| | PAP3 | 0.06-0.07 | 0.06 | 0.06 ab | |
| | ALT3 | 2.23-2.31 | 2.25 | 2.26 de | |
| | CTRL4.5 | 0.10-0.11 | 0.11 | 0.11 bcd | |
| | PAP4.5 | 0.10-0.12 | 0.11 | 0.11 cd | |
| | ALT4.5 | 2.79-3.25 | 3.12 | 3.05 d | |
| | CTRL6 | 0.09-0.11 | 0.10 | 0.10 bc | |
| | PAP6 | 0.08-0.09 | 0.09 | 0.09 abc | |
| | ALT6 | 2.91-3.67 | 3.38 | 3.32 cd | |
| C14:0 | CTRL2 | 3.27-3.40 | 3.29 | 3.32 a | <0.05 |
| | PAP2 | 3.91-4.15 | 3.93 | 4.00 bcd | |
| | ALT2 | 3.67-3.85 | 3.75 | 3.76 abc | |
| | CTRL3 | 3.57-4.05 | 3.57 | 3.73 abc | |
| | PAP3 | 3.59-3.81 | 3.59 | 3.66 ab | |

| | | | | |
|---|---|---|---|---|
| | ALT3 | 3.54-3.73 | 3.58 | 3.62 ab | |
| | CTRL4.5 | 4.27-4.38 | 4.36 | 4.34 d | |
| | PAP4.5 | 4.14-4.44 | 4.34 | 4.31 d | |
| | ALT4.5 | 4.23-4.78 | 4.70 | 4.57 d | |
| | CTRL6 | 3.71-3.82 | 3.75 | 3.76 abc | |
| | PAP6 | 3.96-4.16 | 4.02 | 4.05 bcd | |
| | ALT6 | 3.99-4.38 | 4.36 | 4.24 cd | |
| C15:0 iso | CTRL2 | 0.09-0.13 | 0.12 | 0.11 ab | <0.05 |
| | PAP2 | 0.11-0.12 | 0.12 | 0.12 b | |
| | ALT2 | 0.06-0.12 | 0.07 | 0.08 a | |
| | CTRL3 | 0.06-0.13 | 0.07 | 0.09 ab | |
| | PAP3 | 0.06-0.07 | 0.06 | 0.06 ab | |
| | ALT3 | 0.06-0.06 | 0.06 | 0.06 ab | |
| | CTRL4.5 | 0.10-0.11 | 0.11 | 0.11 ab | |
| | PAP4.5 | 0.10-0.12 | 0.11 | 0.11 ab | |
| | ALT4.5 | 0.10-0.11 | 0.10 | 0.11 ab | |
| | CTRL6 | 0.09-0.11 | 0.10 | 0.10 ab | |
| | PAP6 | 0.08-0.09 | 0.09 | 0.09 ab | |
| | ALT6 | 0.00-0.10 | 0.08 | 0.06 ab | |
| C14:1 | CTRL2 | 0.00-0.07 | 0.06 | 0.04 abc | <0.05 |
| | PAP2 | 0.06-0.06 | 0.06 | 0.06 abc | |
| | ALT2 | 0.06-0.07 | 0.06 | 0.06 abc | |
| | CTRL3 | 0.06-0.07 | 0.06 | 0.07 abc | |
| | PAP3 | 0.00-0.07 | 0.06 | 0.04 abc | |
| | ALT3 | 0.06-0.06 | 0.06 | 0.06 abc | |
| | CTRL4.5 | 0.00-0.00 | 0.00 | 0.00 a | |
| | PAP4.5 | 0.00-0.00 | 0.00 | 0.00 ab | |
| | ALT4.5 | 0.00-0.00 | 0.00 | 0.00 a | |
| | CTRL6 | 0.00-0.10 | 0.00 | 0.03 abc | |
| | PAP6 | 0.08-0.09 | 0.09 | 0.09 bc | |
| | ALT6 | 0.08-0.11 | 0.10 | 0.10 c | |
| C15:0 | CTRL2 | 0.20-0.26 | 0.25 | 0.23 ab | <0.01 |
| | PAP2 | 0.23-0.29 | 0.24 | 0.25 ab | |
| | ALT2 | 0.32-0.35 | 0.33 | 0.33 a | |
| | CTRL3 | 0.21-0.26 | 0.25 | 0.24 a | |
| | PAP3 | 0.19-0.24 | 0.22 | 0.21 a | |
| | ALT3 | 0.29-0.30 | 0.29 | 0.29 a | |
| | CTRL4.5 | 0.30-0.33 | 0.32 | 0.32 ab | |
| | PAP4.5 | 0.23-0.31 | 0.23 | 0.26 b | |
| | ALT4.5 | 0.34-0.42 | 0.41 | 0.39 b | |
| | CTRL6 | 0.19-0.21 | 0.21 | 0.20 a | |
| | PAP6 | 0.17-0.24 | 0.18 | 0.20 a | |
| | ALT6 | 0.33-0.40 | 0.34 | 0.36 a | |
| C16:0 iso | CTRL2 | 0.00-0.00 | 0.00 | 0.00 a | <0.01 |
| | PAP2 | 0.06-0.06 | 0.06 | 0.06 ab | |

| | | | | | |
|---|---|---|---|---|---|
| | ALT2 | 0.00-0.06 | 0.00 | 0.02 a | |
| | CTRL3 | 0.00-0.06 | 0.00 | 0.02 a | |
| | PAP3 | 0.00-0.06 | 0.00 | 0.02 a | |
| | ALT3 | 0.00-0.06 | 0.06 | 0.04 a | |
| | CTRL4.5 | 0.10-0.11 | 0.11 | 0.11 ab | |
| | PAP4.5 | 0.10-0.12 | 0.11 | 0.11 b | |
| | ALT4.5 | 0.10-0.11 | 0.10 | 0.11 b | |
| | CTRL6 | 0.00-0.00 | 0.00 | 0.00 a | |
| | PAP6 | 0.00-0.08 | 0.00 | 0.03 a | |
| | ALT6 | 0.00-0.00 | 0.00 | 0.00 a | |
| C16:0 | CTRL2 | 14.03-14.15 | 14.14 | 14.11 cd | <0.001 |
| | PAP2 | 16.45-16.67 | 16.60 | 16.57 f | |
| | ALT2 | 14.10-14.35 | 14.31 | 14.25 cd | |
| | CTRL3 | 13.97-14.03 | 14.00 | 14.00 bc | |
| | PAP3 | 13.59-13.72 | 13.64 | 13.65 ab | |
| | ALT3 | 13.14-13.45 | 13.34 | 13.31 a | |
| | CTRL4.5 | 13.03-13.25 | 13.08 | 13.12 a | |
| | PAP4.5 | 13.43-13.65 | 13.49 | 13.52 ab | |
| | ALT4.5 | 12.98-13.52 | 13.10 | 13.20 a | |
| | CTRL6 | 14.86-15.24 | 15.09 | 15.06 e | |
| | PAP6 | 15.24-15.33 | 15.30 | 15.29 e | |
| | ALT6 | 14.53-14.87 | 14.79 | 14.73 de | |
| C17:0 iso | CTRL2 | 0.25-0.27 | 0.26 | 0.26 cd | <0.01 |
| | PAP2 | 0.30-0.35 | 0.34 | 0.33 d | |
| | ALT2 | 0.19-0.23 | 0.20 | 0.21 bc | |
| | CTRL3 | 0.21-0.26 | 0.25 | 0.24 bcd | |
| | PAP3 | 0.22-0.25 | 0.24 | 0.24 bcd | |
| | ALT3 | 0.17-0.18 | 0.17 | 0.17 b | |
| | CTRL4.5 | 0.21-0.30 | 0.22 | 0.24 bcd | |
| | PAP4.5 | 0.21-0.23 | 0.23 | 0.22 bcd | |
| | ALT4.5 | 0.21-0.22 | 0.21 | 0.21 bc | |
| | CTRL6 | 0.19-0.21 | 0.21 | 0.20 bc | |
| | PAP6 | 0.17-0.24 | 0.18 | 0.20 bc | |
| | ALT6 | 0.10-0.17 | 0.11 | 0.13 a | |
| C16:1 c9 / C16:1 n7 | CTRL2 | 3.87-4.08 | 3.91 | 3.95 abc | <0.001 |
| | PAP2 | 4.90-5.03 | 4.94 | 4.96 ef | |
| | ALT2 | 3.41-3.66 | 3.58 | 3.55 a | |
| | CTRL3 | 3.94-4.26 | 3.95 | 4.05 bc | |
| | PAP3 | 4.09-4.19 | 4.17 | 4.15 bc | |
| | ALT3 | 3.54-3.68 | 3.64 | 3.62 ab | |
| | CTRL4.5 | 4.36-4.49 | 4.38 | 4.41 cd | |
| | PAP4.5 | 4.45-4.67 | 4.57 | 4.56 de | |
| | ALT4.5 | 3.82-4.26 | 4.14 | 4.07 bc | |
| | CTRL6 | 4.78-4.99 | 4.84 | 4.87 ef | |
| | PAP6 | 5.20-5.30 | 5.24 | 5.25 f | |

| | | | | | |
|---|---|---|---|---|---|
| | ALT6 | 4.57-4.88 | 4.82 | 4.75 de | |
| C17:0 | CTRL2 | 0.27-0.34 | 0.31 | 0.31 e | <0.001 |
| | PAP2 | 0.29-0.30 | 0.29 | 0.29 e | |
| | ALT2 | 0.17-0.20 | 0.19 | 0.19 abc | |
| | CTRL3 | 0.25-0.28 | 0.26 | 0.26 de | |
| | PAP3 | 0.22-0.25 | 0.24 | 0.24 d | |
| | ALT3 | 0.17-0.18 | 0.17 | 0.17 a | |
| | CTRL4.5 | 0.20-0.22 | 0.21 | 0.21 abcd | |
| | PAP4.5 | 0.21-0.23 | 0.23 | 0.22 cd | |
| | ALT4.5 | 0.21-0.22 | 0.21 | 0.21 bcd | |
| | CTRL6 | 0.11-0.21 | 0.19 | 0.17 ab | |
| | PAP6 | 0.16-0.18 | 0.17 | 0.17 a | |
| | ALT6 | 0.10-0.17 | 0.11 | 0.13 a | |
| C16:3 n4 | CTRL2 | 0.00-0.07 | 0.06 | 0.04 ab | <0.001 |
| | PAP2 | 0.06-0.06 | 0.06 | 0.06 ab | |
| | ALT2 | 0.06-0.07 | 0.06 | 0.06 ab | |
| | CTRL3 | 0.06-0.07 | 0.06 | 0.07 abc | |
| | PAP3 | 0.06-0.07 | 0.06 | 0.06 ab | |
| | ALT3 | 0.06-0.06 | 0.06 | 0.06 a | |
| | CTRL4.5 | 0.20-0.22 | 0.21 | 0.21 f | |
| | PAP4.5 | 0.12-0.23 | 0.21 | 0.18 ef | |
| | ALT4.5 | 0.10-0.11 | 0.10 | 0.11 def | |
| | CTRL6 | 0.09-0.11 | 0.10 | 0.10 de | |
| | PAP6 | 0.08-0.09 | 0.09 | 0.09 bcd | |
| | ALT6 | 0.08-0.11 | 0.10 | 0.10 cde | |
| C17:2 c9,10 | CTRL2 | 0.06-0.09 | 0.07 | 0.07 abcd | <0.01 |
| | PAP2 | 0.06-0.11 | 0.06 | 0.08 abcd | |
| | ALT2 | 0.06-0.07 | 0.06 | 0.06 ab | |
| | CTRL3 | 0.06-0.07 | 0.06 | 0.07 abc | |
| | PAP3 | 0.06-0.07 | 0.06 | 0.06 ab | |
| | ALT3 | 0.06-0.06 | 0.06 | 0.06 a | |
| | CTRL4.5 | 0.10-0.11 | 0.11 | 0.11 bcde | |
| | PAP4.5 | 0.10-0.12 | 0.11 | 0.11 de | |
| | ALT4.5 | 0.10-0.22 | 0.10 | 0.14 cde | |
| | CTRL6 | 0.09-0.11 | 0.10 | 0.10 bcde | |
| | PAP6 | 0.08-0.09 | 0.09 | 0.09 abcd | |
| | ALT6 | 0.20-0.25 | 0.23 | 0.23 e | |
| C17:1 c10 | CTRL2 | 0.43-0.47 | 0.43 | 0.44 de | <0.001 |
| | PAP2 | 0.48-0.53 | 0.52 | 0.51 ef | |
| | ALT2 | 0.38-0.40 | 0.39 | 0.39 cd | |
| | CTRL3 | 0.44-0.50 | 0.45 | 0.46 de | |
| | PAP3 | 0.50-0.58 | 0.54 | 0.54 f | |
| | ALT3 | 0.46-0.47 | 0.46 | 0.46 def | |
| | CTRL4.5 | 0.00-0.00 | 0.00 | 0.00 a | |
| | PAP4.5 | 0.00-0.00 | 0.00 | 0.00 a | |

| | | | | | |
|---|---|---|---|---|---|
| | ALT4.5 | 0.00-0.00 | 0.00 | 0.00 a | |
| | CTRL6 | 0.09-0.11 | 0.10 | 0.10 bc | |
| | PAP6 | 0.08-0.09 | 0.09 | 0.09 b | |
| | ALT6 | 0.08-0.11 | 0.10 | 0.10 bc | |
| C16:4 n1 | CTRL2 | 0.09-0.13 | 0.12 | 0.11 bc | <0.001 |
| | PAP2 | 0.06-0.06 | 0.06 | 0.06 b | |
| | ALT2 | 0.23-0.26 | 0.25 | 0.25 a | |
| | CTRL3 | 0.07-0.13 | 0.13 | 0.11 bc | |
| | PAP3 | 0.06-0.12 | 0.07 | 0.08 bc | |
| | ALT3 | 0.17-0.18 | 0.17 | 0.17 c | |
| | CTRL4.5 | 0.00-0.00 | 0.00 | 0.00 a | |
| | PAP4.5 | 0.00-0.00 | 0.00 | 0.00 a | |
| | ALT4.5 | 0.00-0.00 | 0.00 | 0.00 a | |
| | CTRL6 | 0.00-0.00 | 0.00 | 0.00 a | |
| | PAP6 | 0.00-0.00 | 0.00 | 0.00 a | |
| | ALT6 | 0.00-0.00 | 0.00 | 0.00 a | |
| C16:2n4 | CTRL2 | 0.00-0.00 | 0.00 | 0.00 a | <0.01 |
| | PAP2 | 0.00-0.00 | 0.00 | 0.00 a | |
| | ALT2 | 0.00-0.00 | 0.00 | 0.00 a | |
| | CTRL3 | 0.00-0.00 | 0.00 | 0.00 a | |
| | PAP3 | 0.00-0.00 | 0.00 | 0.00 ab | |
| | ALT3 | 0.00-0.00 | 0.00 | 0.00 a | |
| | CTRL4.5 | 0.44-0.53 | 0.50 | 0.49 b | |
| | PAP4.5 | 0.41-0.57 | 0.47 | 0.48 b | |
| | ALT4.5 | 0.41-0.45 | 0.42 | 0.43 b | |
| | CTRL6 | 0.41-0.53 | 0.47 | 0.47 b | |
| | PAP6 | 0.44-0.52 | 0.49 | 0.48 b | |
| | ALT6 | 0.42-0.50 | 0.46 | 0.46 b | |
| C18:0 | CTRL2 | 3.10-3.20 | 3.15 | 3.15 cd | <0.01 |
| | PAP2 | 3.98-4.08 | 3.99 | 4.02 d | |
| | ALT2 | 2.81-3.10 | 2.94 | 2.95 bcd | |
| | CTRL3 | 2.84-3.38 | 3.25 | 3.16 bcd | |
| | PAP3 | 2.88-3.08 | 3.05 | 3.00 bcd | |
| | ALT3 | 2.85-3.00 | 2.91 | 2.92 bcd | |
| | CTRL4.5 | 2.41-2.48 | 2.46 | 2.45 a | |
| | PAP4.5 | 2.57-2.79 | 2.63 | 2.66 ab | |
| | ALT4.5 | 2.18-2.79 | 2.35 | 2.44 a | |
| | CTRL6 | 2.65-2.91 | 2.78 | 2.78 abc | |
| | PAP6 | 2.77-2.91 | 2.80 | 2.83 abc | |
| | ALT6 | 2.39-2.66 | 2.41 | 2.48 a | |
| C18:1 t9 | CTRL2 | 0.13-0.19 | 0.17 | 0.16 abc | <0.01 |
| | PAP2 | 0.17-0.18 | 0.18 | 0.18 bc | |
| | ALT2 | 0.17-0.20 | 0.19 | 0.19 bc | |
| | CTRL3 | 0.19-0.21 | 0.19 | 0.20 c | |
| | PAP3 | 0.14-0.19 | 0.18 | 0.17 bc | |

| | | | | | |
|---|---|---|---|---|---|
| | ALT3 | 0.17-0.18 | 0.17 | 0.17 bc | |
| | CTRL4.5 | 0.00-0.00 | 0.00 | 0.00 a | |
| | PAP4.5 | 0.00-0.10 | 0.00 | 0.03 ab | |
| | ALT4.5 | 0.00-0.00 | 0.00 | 0.00 a | |
| | CTRL6 | 0.00-0.10 | 0.00 | 0.03 a | |
| | PAP6 | 0.00-0.08 | 0.00 | 0.03 a | |
| | ALT6 | 0.00-0.10 | 0.00 | 0.03 a | |
| C18:1 c9 / C18:1 n9 | CTRL2 | 34.01-35.09 | 34.95 | 34.68 ef | <0.01 |
| | PAP2 | 27.08-27.77 | 27.69 | 27.51 a | |
| | ALT2 | 31.53-32.62 | 32.31 | 32.15 abcd | |
| | CTRL3 | 35.32-37.05 | 36.94 | 36.44 f | |
| | PAP3 | 32.93-33.73 | 33.48 | 33.38 bcde | |
| | ALT3 | 34.02-34.41 | 34.15 | 34.20 def | |
| | CTRL4.5 | 33.97-34.29 | 34.06 | 34.11 def | |
| | PAP4.5 | 32.71-34.13 | 32.80 | 33.21 bcde | |
| | ALT4.5 | 33.13-34.88 | 33.15 | 33.72 cdef | |
| | CTRL6 | 33.55-34.21 | 33.68 | 33.81 def | |
| | PAP6 | 31.62-32.16 | 31.64 | 31.81 ab | |
| | ALT6 | 31.31-32.89 | 31.64 | 31.95 abc | |
| C18:1 n7 / C18:1 c11 | CTRL2 | 3.41-3.57 | 3.54 | 3.51 i | <0.001 |
| | PAP2 | 3.27-3.33 | 3.27 | 3.29 g | |
| | ALT2 | 3.00-3.10 | 3.07 | 3.06 cde | |
| | CTRL3 | 3.55-3.63 | 3.57 | 3.59 i | |
| | PAP3 | 3.35-3.40 | 3.38 | 3.38 h | |
| | ALT3 | 3.14-3.20 | 3.18 | 3.17 efg | |
| | CTRL4.5 | 2.96-2.99 | 2.97 | 2.97 bcd | |
| | PAP4.5 | 2.86-2.92 | 2.90 | 2.89 ab | |
| | ALT4.5 | 2.60-2.69 | 2.68 | 2.66 a | |
| | CTRL6 | 3.18-3.19 | 3.19 | 3.19 fg | |
| | PAP6 | 3.06-3.17 | 3.10 | 3.11 def | |
| | ALT6 | 2.87-2.99 | 2.89 | 2.91 abc | |
| C19:0 | CTRL2 | 0.06-0.09 | 0.07 | 0.07 b | <0.05 |
| | PAP2 | 0.06-0.06 | 0.06 | 0.06 ab | |
| | ALT2 | 0.06-0.07 | 0.06 | 0.06 ab | |
| | CTRL3 | 0.06-0.07 | 0.06 | 0.07 ab | |
| | PAP3 | 0.06-0.07 | 0.06 | 0.06 ab | |
| | ALT3 | 0.06-0.06 | 0.06 | 0.06 ab | |
| | CTRL4.5 | 0.00-0.11 | 0.10 | 0.07 ab | |
| | PAP4.5 | 0.00-0.12 | 0.10 | 0.07 ab | |
| | ALT4.5 | 0.00-0.00 | 0.00 | 0.00 a | |
| | CTRL6 | 0.00-0.00 | 0.00 | 0.00 ab | |
| | PAP6 | 0.00-0.08 | 0.00 | 0.03 ab | |
| | ALT6 | 0.00-0.00 | 0.00 | 0.00 ab | |
| C19:2 c9,10 | CTRL2 | 0.00-0.00 | 0.00 | 0.00 a | <0.001 |
| | PAP2 | 0.00-0.00 | 0.00 | 0.00 a | |

| | | | | | |
|---|---|---|---|---|---|
| | ALT2 | 0.00-0.00 | 0.00 | 0.00 a | |
| | CTRL3 | 0.00-0.00 | 0.00 | 0.00 a | |
| | PAP3 | 0.00-0.00 | 0.00 | 0.00 ab | |
| | ALT3 | 0.00-0.00 | 0.00 | 0.00 a | |
| | CTRL4.5 | 0.99-1.07 | 0.99 | 1.02 bcd | |
| | PAP4.5 | 0.83-1.05 | 0.91 | 0.93 bc | |
| | ALT4.5 | 0.83-1.04 | 1.01 | 0.96 bc | |
| | CTRL6 | 1.22-1.38 | 1.24 | 1.28 cd | |
| | PAP6 | 1.23-1.39 | 1.31 | 1.31 d | |
| | ALT6 | 1.16-1.39 | 1.38 | 1.31 d | |
| C18:2 c9,12 / C18:2 n6 | CTRL2 | 14.48-14.63 | 14.59 | 14.56 bcd | <0.001 |
| | PAP2 | 13.84-14.04 | 13.97 | 13.95 ab | |
| | ALT2 | 14.71-15.08 | 14.73 | 14.84 cde | |
| | CTRL3 | 14.54-15.28 | 14.59 | 14.80 cd | |
| | PAP3 | 15.37-15.67 | 15.48 | 15.51 e | |
| | ALT3 | 15.36-15.53 | 15.36 | 15.42 de | |
| | CTRL4.5 | 13.68-14.13 | 14.10 | 13.97 abc | |
| | PAP4.5 | 13.24-13.90 | 13.60 | 13.58 ab | |
| | ALT4.5 | 13.29-13.66 | 13.31 | 13.42 a | |
| | CTRL6 | 14.62-15.04 | 14.76 | 14.80 cd | |
| | PAP6 | 13.69-14.16 | 13.83 | 13.90 ab | |
| | ALT6 | 13.62-14.11 | 13.73 | 13.82 ab | |
| C18:2 n4 | CTRL2 | 0.00-0.00 | 0.00 | 0.00 a | <0.001 |
| | PAP2 | 0.00-0.00 | 0.00 | 0.00 a | |
| | ALT2 | 0.00-0.00 | 0.00 | 0.00 a | |
| | CTRL3 | 0.00-0.00 | 0.00 | 0.00 a | |
| | PAP3 | 0.00-0.00 | 0.00 | 0.00 a | |
| | ALT3 | 0.00-0.00 | 0.00 | 0.00 a | |
| | CTRL4.5 | 0.10-0.11 | 0.11 | 0.11 b | |
| | PAP4.5 | 0.10-0.12 | 0.11 | 0.11 b | |
| | ALT4.5 | 0.10-0.21 | 0.11 | 0.14 b | |
| | CTRL6 | 0.11-0.21 | 0.19 | 0.17 b | |
| | PAP6 | 0.16-0.18 | 0.17 | 0.17 b | |
| | ALT6 | 0.11-0.20 | 0.17 | 0.16 b | |
| C20:0 | CTRL2 | 0.31-0.34 | 0.33 | 0.33 c | <0.05 |
| | PAP2 | 0.23-0.24 | 0.23 | 0.24 abc | |
| | ALT2 | 0.26-0.32 | 0.29 | 0.29 abc | |
| | CTRL3 | 0.28-0.38 | 0.38 | 0.35 c | |
| | PAP3 | 0.29-0.31 | 0.30 | 0.30 abc | |
| | ALT3 | 0.30-0.35 | 0.34 | 0.33 bc | |
| | CTRL4.5 | 0.30-0.33 | 0.32 | 0.32 abc | |
| | PAP4.5 | 0.23-0.31 | 0.23 | 0.26 abc | |
| | ALT4.5 | 0.21-0.31 | 0.22 | 0.25 abc | |
| | CTRL6 | 0.21-0.28 | 0.21 | 0.23 a | |
| | PAP6 | 0.17-0.26 | 0.24 | 0.23 ab | |

| | | | | | |
|---|---|---|---|---|---|
| | ALT6 | 0.20-0.25 | 0.23 | 0.23 a | |
| C18:3 c6,9,12 gamma / C18:3 n6 | CTRL2 | 0.09-0.13 | 0.12 | 0.11 ab | <0.05 |
| | PAP2 | 0.17-0.18 | 0.18 | 0.18 b | |
| | ALT2 | 0.12-0.13 | 0.13 | 0.12 ab | |
| | CTRL3 | 0.07-0.13 | 0.13 | 0.11 ab | |
| | PAP3 | 0.12-0.14 | 0.13 | 0.13 ab | |
| | ALT3 | 0.11-0.12 | 0.12 | 0.12 ab | |
| | CTRL4.5 | 0.10-0.11 | 0.11 | 0.11 a | |
| | PAP4.5 | 0.10-0.12 | 0.11 | 0.11 ab | |
| | ALT4.5 | 0.10-0.11 | 0.10 | 0.11 ab | |
| | CTRL6 | 0.09-0.11 | 0.10 | 0.10 a | |
| | PAP6 | 0.08-0.09 | 0.09 | 0.09 a | |
| | ALT6 | 0.08-0.11 | 0.10 | 0.10 a | |
| C18:3 n4 | CTRL2 | 0.00-0.00 | 0.00 | 0.00 a | <0.001 |
| | PAP2 | 0.00-0.06 | 0.06 | 0.04 ab | |
| | ALT2 | 0.00-0.00 | 0.00 | 0.00 a | |
| | CTRL3 | 0.00-0.00 | 0.00 | 0.00 a | |
| | PAP3 | 0.00-0.00 | 0.00 | 0.00 a | |
| | ALT3 | 0.00-0.00 | 0.00 | 0.00 a | |
| | CTRL4.5 | 0.10-0.11 | 0.11 | 0.11 bc | |
| | PAP4.5 | 0.10-0.12 | 0.11 | 0.11 bc | |
| | ALT4.5 | 0.10-0.11 | 0.10 | 0.11 bc | |
| | CTRL6 | 0.10-0.19 | 0.11 | 0.13 bc | |
| | PAP6 | 0.17-0.26 | 0.24 | 0.23 b | |
| | ALT6 | 0.08-0.11 | 0.10 | 0.10 bc | |
| C20:1 c11 / C20:1 n9 | CTRL2 | 1.53-1.60 | 1.55 | 1.56 gh | <0.001 |
| | PAP2 | 2.22-2.36 | 2.35 | 2.31 h | |
| | ALT2 | 1.04-1.14 | 1.10 | 1.09 def | |
| | CTRL3 | 1.21-1.47 | 1.44 | 1.37 efg | |
| | PAP3 | 1.37-1.57 | 1.50 | 1.48 fgh | |
| | ALT3 | 0.98-1.03 | 1.01 | 1.01 d | |
| | CTRL4.5 | 0.75-0.79 | 0.77 | 0.77 b | |
| | PAP4.5 | 1.03-1.14 | 1.05 | 1.07 de | |
| | ALT4.5 | 0.62-0.72 | 0.67 | 0.67 ab | |
| | CTRL6 | 0.62-0.66 | 0.64 | 0.64 ab | |
| | PAP6 | 0.87-0.97 | 0.90 | 0.91 c | |
| | ALT6 | 0.46-0.58 | 0.50 | 0.51 a | |
| C18:3 c9,12,15 alpha / C18:3 n3 | CTRL2 | 4.00-4.08 | 4.03 | 4.04 fgh | <0.001 |
| | PAP2 | 2.70-2.75 | 2.72 | 2.72 a | |
| | ALT2 | 3.73-3.98 | 3.81 | 3.84 defg | |
| | CTRL3 | 3.88-4.41 | 3.89 | 4.06 efgh | |
| | PAP3 | 4.19-4.39 | 4.22 | 4.26 gh | |
| | ALT3 | 4.21-5.20 | 4.27 | 4.56 h | |
| | CTRL4.5 | 3.85-3.94 | 3.87 | 3.88 defgh | |
| | PAP4.5 | 3.62-3.89 | 3.74 | 3.75 cdef | |

| | | | | | |
|---|---|---|---|---|---|
| | ALT4.5 | 3.41-3.84 | 3.70 | 3.65 cde | |
| | CTRL6 | 3.37-3.50 | 3.50 | 3.46 bcd | |
| | PAP6 | 3.26-3.41 | 3.34 | 3.34 bc | |
| | ALT6 | 3.16-3.28 | 3.21 | 3.22 b | |
| C21:0 | CTRL2 | 0.00-0.00 | 0.00 | 0.00 | 0.504 |
| | PAP2 | 0.00-0.00 | 0.00 | 0.00 | |
| | ALT2 | 0.00-0.06 | 0.02 | 0.02 | |
| | CTRL3 | 0.00-0.06 | 0.02 | 0.02 | |
| | PAP3 | 0.00-0.00 | 0.00 | 0.00 | |
| | ALT3 | 0.00-0.00 | 0.00 | 0.00 | |
| | CTRL4.5 | 0.00-0.00 | 0.00 | 0.00 | |
| | PAP4.5 | 0.00-0.00 | 0.00 | 0.00 | |
| | ALT4.5 | 0.00-0.00 | 0.00 | 0.00 | |
| | CTRL6 | 0.00-0.00 | 0.00 | 0.00 | |
| | PAP6 | 0.00-0.00 | 0.00 | 0.00 | |
| | ALT6 | 0.00-0.00 | 0.00 | 0.00 | |
| C18:4 n3 | CTRL2 | 0.06-0.09 | 0.07 | 0.07 a | <0.001 |
| | PAP2 | 0.11-0.12 | 0.12 | 0.12 abc | |
| | ALT2 | 0.06-0.07 | 0.06 | 0.06 a | |
| | CTRL3 | 0.06-0.13 | 0.07 | 0.09 ab | |
| | PAP3 | 0.06-0.07 | 0.06 | 0.06 a | |
| | ALT3 | 0.11-0.12 | 0.12 | 0.12 ab | |
| | CTRL4.5 | 1.20-1.39 | 1.29 | 1.29 e | |
| | PAP4.5 | 1.14-1.49 | 1.17 | 1.26 e | |
| | ALT4.5 | 0.93-1.04 | 1.01 | 0.99 e | |
| | CTRL6 | 0.56-0.64 | 0.62 | 0.61 cd | |
| | PAP6 | 0.70-0.79 | 0.73 | 0.74 d | |
| | ALT6 | 0.50-0.60 | 0.57 | 0.56 bcd | |
| C20:2 c11,14 | CTRL2 | 0.13-0.19 | 0.17 | 0.16 bcd | <0.01 |
| | PAP2 | 0.52-0.54 | 0.53 | 0.53 e | |
| | ALT2 | 0.12-0.13 | 0.13 | 0.12 abcd | |
| | CTRL3 | 0.14-0.19 | 0.19 | 0.17 bcde | |
| | PAP3 | 0.22-0.25 | 0.24 | 0.24 cde | |
| | ALT3 | 0.11-0.12 | 0.12 | 0.12 abc | |
| | CTRL4.5 | 0.11-0.53 | 0.30 | 0.31 bcde | |
| | PAP4.5 | 0.31-0.46 | 0.35 | 0.37 de | |
| | ALT4.5 | 0.10-0.21 | 0.11 | 0.14 abcd | |
| | CTRL6 | 0.09-0.11 | 0.10 | 0.10 a | |
| | PAP6 | 0.18-0.26 | 0.24 | 0.23 bcde | |
| | ALT6 | 0.08-0.11 | 0.10 | 0.10 ab | |
| C22:0 | CTRL2 | 0.17-0.20 | 0.19 | 0.19 | 0.066 |
| | PAP2 | 0.11-0.12 | 0.12 | 0.12 | |
| | ALT2 | 0.13-0.19 | 0.17 | 0.16 | |
| | CTRL3 | 0.14-0.25 | 0.19 | 0.19 | |

| | | | | | |
|---|---|---|---|---|---|
| | PAP3 | 0.13-0.18 | 0.14 | 0.15 | |
| | ALT3 | 0.17-0.18 | 0.17 | 0.17 | |
| | CTRL4.5 | 0.11-0.21 | 0.20 | 0.17 | |
| | PAP4.5 | 0.11-0.31 | 0.12 | 0.18 | |
| | ALT4.5 | 0.11-0.31 | 0.21 | 0.21 | |
| | CTRL6 | 0.10-0.19 | 0.11 | 0.13 | |
| | PAP6 | 0.08-0.09 | 0.09 | 0.09 | |
| | ALT6 | 0.08-0.11 | 0.10 | 0.10 | |
| C20:3 c8,11,14 | CTRL2 | 0.50-0.53 | 0.51 | 0.51 d | <0.001 |
| | PAP2 | 1.46-1.57 | 1.55 | 1.53 d | |
| | ALT2 | 0.26-0.32 | 0.29 | 0.29 bc | |
| | CTRL3 | 0.36-0.45 | 0.44 | 0.41 cd | |
| | PAP3 | 0.43-0.50 | 0.48 | 0.47 cd | |
| | ALT3 | 0.11-0.12 | 0.12 | 0.12 ab | |
| | CTRL4.5 | 0.00-0.11 | 0.10 | 0.07 a | |
| | PAP4.5 | 0.10-0.12 | 0.11 | 0.11 ab | |
| | ALT4.5 | 0.00-0.11 | 0.10 | 0.07 a | |
| | CTRL6 | 0.09-0.11 | 0.10 | 0.10 a | |
| | PAP6 | 0.08-0.09 | 0.09 | 0.09 a | |
| | ALT6 | 0.08-0.11 | 0.10 | 0.10 a | |
| C22:1 n11 | CTRL2 | 0.00-0.00 | 0.00 | 0.00 a | <0.001 |
| | PAP2 | 0.00-0.00 | 0.00 | 0.00 a | |
| | ALT2 | 0.00-0.00 | 0.00 | 0.00 a | |
| | CTRL3 | 0.00-0.00 | 0.00 | 0.00 a | |
| | PAP3 | 0.00-0.00 | 0.00 | 0.00 a | |
| | ALT3 | 0.00-0.00 | 0.00 | 0.00 a | |
| | CTRL4.5 | 0.21-0.30 | 0.22 | 0.24 cd | |
| | PAP4.5 | 0.46-0.52 | 0.47 | 0.48 d | |
| | ALT4.5 | 0.10-0.11 | 0.10 | 0.11 b | |
| | CTRL6 | 0.19-0.21 | 0.21 | 0.20 bc | |
| | PAP6 | 0.52-0.57 | 0.53 | 0.54 e | |
| | ALT6 | 0.11-0.25 | 0.20 | 0.19 bcd | |
| C22:1 c13 / C22:1 n9 | CTRL2 | 0.53-0.60 | 0.56 | 0.56 de | <0.001 |
| | PAP2 | 0.73-0.76 | 0.75 | 0.74 f | |
| | ALT2 | 0.59-0.63 | 0.63 | 0.62 e | |
| | CTRL3 | 0.50-0.51 | 0.50 | 0.50 c | |
| | PAP3 | 0.57-0.60 | 0.58 | 0.58 de | |
| | ALT3 | 0.51-0.53 | 0.52 | 0.52 d | |
| | CTRL4.5 | 0.20-0.22 | 0.21 | 0.21 abc | |
| | PAP4.5 | 0.21-0.23 | 0.23 | 0.22 bc | |
| | ALT4.5 | 0.10-0.21 | 0.11 | 0.14 a | |
| | CTRL6 | 0.10-0.19 | 0.11 | 0.13 a | |
| | PAP6 | 0.16-0.18 | 0.17 | 0.17 ab | |
| | ALT6 | 0.10-0.17 | 0.11 | 0.13 a | |
| C20:3 c11,14,17 | CTRL2 | 0.06-0.09 | 0.07 | 0.07 bcd | <0.001 |

| | | | | | |
|---|---|---|---|---|---|
| | PAP2 | 0.17-0.18 | 0.18 | 0.18 f | |
| | ALT2 | 0.06-0.07 | 0.06 | 0.06 bc | |
| | CTRL3 | 0.06-0.07 | 0.06 | 0.07 bc | |
| | PAP3 | 0.12-0.14 | 0.13 | 0.13 e | |
| | ALT3 | 0.06-0.06 | 0.06 | 0.06 bc | |
| | CTRL4.5 | 0.10-0.11 | 0.11 | 0.11 cde | |
| | PAP4.5 | 0.10-0.12 | 0.11 | 0.11 de | |
| | ALT4.5 | 0.00-0.10 | 0.10 | 0.07 bcd | |
| | CTRL6 | 0.00-0.00 | 0.00 | 0.00 ab | |
| | PAP6 | 0.08-0.09 | 0.09 | 0.09 bcd | |
| | ALT6 | 0.00-0.00 | 0.00 | 0.00 a | |
| C20:4 c5,8,11,14 / C20:4 n6 | CTRL2 | 0.25-0.27 | 0.26 | 0.26 cd | <0.001 |
| | PAP2 | 0.23-0.30 | 0.29 | 0.27 cde | |
| | ALT2 | 0.13-0.19 | 0.17 | 0.16 a | |
| | CTRL3 | 0.21-0.26 | 0.25 | 0.24 bcd | |
| | PAP3 | 0.19-0.24 | 0.22 | 0.21 abc | |
| | ALT3 | 0.17-0.18 | 0.17 | 0.17 ab | |
| | CTRL4.5 | 0.40-0.44 | 0.43 | 0.42 def | |
| | PAP4.5 | 0.41-0.47 | 0.46 | 0.45 efg | |
| | ALT4.5 | 0.41-0.45 | 0.42 | 0.43 ef | |
| | CTRL6 | 0.62-0.66 | 0.64 | 0.64 h | |
| | PAP6 | 0.57-0.62 | 0.61 | 0.60 gh | |
| | ALT6 | 0.50-0.58 | 0.57 | 0.55 fgh | |
| C23:0 | CTRL2 | 0.06-0.09 | 0.07 | 0.07 d | <0.01 |
| | PAP2 | 0.06-0.06 | 0.06 | 0.06 cd | |
| | ALT2 | 0.06-0.07 | 0.06 | 0.06 cd | |
| | CTRL3 | 0.06-0.07 | 0.06 | 0.07 d | |
| | PAP3 | 0.06-0.07 | 0.06 | 0.06 d | |
| | ALT3 | 0.06-0.06 | 0.06 | 0.06 bcd | |
| | CTRL4.5 | 0.00-0.10 | 0.00 | 0.03 abcd | |
| | PAP4.5 | 0.00-0.00 | 0.00 | 0.00 abc | |
| | ALT4.5 | 0.00-0.00 | 0.00 | 0.00 a | |
| | CTRL6 | 0.00-0.00 | 0.00 | 0.00 ab | |
| | PAP6 | 0.00-0.00 | 0.00 | 0.00 abcd | |
| | ALT6 | 0.00-0.00 | 0.00 | 0.00 a | |
| C20:4 n3 | CTRL2 | 0.00-0.00 | 0.00 | 0.00 a | <0.001 |
| | PAP2 | 0.00-0.00 | 0.00 | 0.00 a | |
| | ALT2 | 0.00-0.00 | 0.00 | 0.00 a | |
| | CTRL3 | 0.00-0.00 | 0.00 | 0.00 a | |
| | PAP3 | 0.00-0.00 | 0.00 | 0.00 ab | |
| | ALT3 | 0.00-0.00 | 0.00 | 0.00 a | |
| | CTRL4.5 | 0.30-0.33 | 0.32 | 0.32 cd | |
| | PAP4.5 | 0.34-0.41 | 0.35 | 0.37 d | |
| | ALT4.5 | 0.21-0.22 | 0.21 | 0.21 bc | |
| | CTRL6 | 0.19-0.21 | 0.21 | 0.20 bc | |

| | | | | | |
|---|---|---|---|---|---|
| | PAP6 | 0.33-0.35 | 0.35 | 0.34 d | |
| | ALT6 | 0.20-0.25 | 0.23 | 0.23 bc | |
| C22:2 c13,16 | CTRL2 | 0.06-0.09 | 0.07 | 0.07 abc | <0.05 |
| | PAP2 | 0.06-0.11 | 0.06 | 0.08 abc | |
| | ALT2 | 0.00-0.00 | 0.00 | 0.00 a | |
| | CTRL3 | 0.00-0.06 | 0.00 | 0.02 abc | |
| | PAP3 | 0.06-0.07 | 0.06 | 0.06 abc | |
| | ALT3 | 0.00-0.06 | 0.06 | 0.04 abc | |
| | CTRL4.5 | 0.00-0.11 | 0.10 | 0.07 abc | |
| | PAP4.5 | 0.10-0.12 | 0.11 | 0.11 c | |
| | ALT4.5 | 0.00-0.10 | 0.00 | 0.03 abc | |
| | CTRL6 | 0.00-0.00 | 0.00 | 0.00 ab | |
| | PAP6 | 0.08-0.09 | 0.09 | 0.09 bc | |
| | ALT6 | 0.00-0.00 | 0.00 | 0.00 ab | |
| C24:0 | CTRL2 | 0.12-0.17 | 0.13 | 0.14 de | <0.01 |
| | PAP2 | 0.11-0.12 | 0.12 | 0.12 de | |
| | ALT2 | 0.12-0.19 | 0.13 | 0.15 de | |
| | CTRL3 | 0.19-0.21 | 0.19 | 0.20 e | |
| | PAP3 | 0.12-0.14 | 0.13 | 0.13 de | |
| | ALT3 | 0.11-0.12 | 0.12 | 0.12 de | |
| | CTRL4.5 | 0.10-0.11 | 0.11 | 0.11 bcd | |
| | PAP4.5 | 0.10-0.12 | 0.11 | 0.11 cde | |
| | ALT4.5 | 0.10-0.22 | 0.21 | 0.18 de | |
| | CTRL6 | 0.00-0.00 | 0.00 | 0.00 a | |
| | PAP6 | 0.00-0.09 | 0.08 | 0.06 abc | |
| | ALT6 | 0.00-0.08 | 0.00 | 0.03 ab | |
| C20:5 c5,8,11,14,17 EPA / C20:5 n3 | CTRL2 | 6.07-6.38 | 6.15 | 6.20 abc | <0.001 |
| | PAP2 | 6.72-6.90 | 6.78 | 6.80 bcd | |
| | ALT2 | 6.01-6.33 | 6.12 | 6.15 ab | |
| | CTRL3 | 5.07-5.83 | 5.29 | 5.40 a | |
| | PAP3 | 6.70-7.05 | 6.73 | 6.83 cd | |
| | ALT3 | 6.11-6.22 | 6.12 | 6.15 ab | |
| | CTRL4.5 | 6.84-7.05 | 7.01 | 6.97 cde | |
| | PAP4.5 | 6.62-7.01 | 6.97 | 6.87 cd | |
| | ALT4.5 | 6.50-7.37 | 6.94 | 6.94 cd | |
| | CTRL6 | 7.69-8.17 | 7.72 | 7.86 ef | |
| | PAP6 | 8.11-8.39 | 8.31 | 8.27 f | |
| | ALT6 | 7.64-8.06 | 7.91 | 7.87 def | |
| C24:1 c15 / C24:1 n9 | CTRL2 | 0.26-0.33 | 0.31 | 0.30 f | <0.001 |
| | PAP2 | 0.29-0.30 | 0.29 | 0.29 ef | |
| | ALT2 | 0.17-0.20 | 0.19 | 0.19 abcd | |
| | CTRL3 | 0.21-0.31 | 0.26 | 0.26 def | |
| | PAP3 | 0.18-0.22 | 0.19 | 0.19 bcde | |
| | ALT3 | 0.17-0.18 | 0.17 | 0.17 abc | |
| | CTRL4.5 | 0.20-0.22 | 0.21 | 0.21 cdef | |

| | | | | | |
|---|---|---|---|---|---|
| | PAP4.5 | 0.21-0.23 | 0.23 | 0.22 def | |
| | ALT4.5 | 0.00-0.11 | 0.10 | 0.07 a | |
| | CTRL6 | 0.09-0.11 | 0.10 | 0.10 a | |
| | PAP6 | 0.09-0.18 | 0.16 | 0.14 ab | |
| | ALT6 | 0.08-0.11 | 0.10 | 0.10 a | |
| C24:2 n6 | CTRL2 | 0.00-0.00 | 0.00 | 0.00 ab | <0.01 |
| | PAP2 | 0.00-0.00 | 0.00 | 0.00 abcd | |
| | ALT2 | 0.00-0.00 | 0.00 | 0.00 a | |
| | CTRL3 | 0.00-0.00 | 0.00 | 0.00 abc | |
| | PAP3 | 0.00-0.00 | 0.00 | 0.00 abcde | |
| | ALT3 | 0.00-0.00 | 0.00 | 0.00 a | |
| | CTRL4.5 | 0.00-0.11 | 0.10 | 0.07 bcde | |
| | PAP4.5 | 0.10-0.12 | 0.11 | 0.11 e | |
| | ALT4.5 | 0.00-0.10 | 0.00 | 0.03 abcde | |
| | CTRL6 | 0.09-0.11 | 0.10 | 0.10 de | |
| | PAP6 | 0.08-0.09 | 0.09 | 0.09 bcde | |
| | ALT6 | 0.08-0.11 | 0.10 | 0.10 cde | |
| C21:5n3 | CTRL2 | 0.19-0.26 | 0.20 | 0.21 abc | <0.01 |
| | PAP2 | 0.29-0.30 | 0.29 | 0.29 cd | |
| | ALT2 | 0.17-0.20 | 0.19 | 0.19 a | |
| | CTRL3 | 0.19-0.21 | 0.19 | 0.20 ab | |
| | PAP3 | 0.24-0.29 | 0.25 | 0.26 bcd | |
| | ALT3 | 0.23-0.24 | 0.23 | 0.23 abcd | |
| | CTRL4.5 | 0.20-0.22 | 0.21 | 0.21 abc | |
| | PAP4.5 | 0.21-0.23 | 0.23 | 0.22 abcd | |
| | ALT4.5 | 0.21-0.22 | 0.21 | 0.21 abc | |
| | CTRL6 | 0.21-0.32 | 0.28 | 0.27 abcd | |
| | PAP6 | 0.26-0.35 | 0.33 | 0.31 d | |
| | ALT6 | 0.23-0.30 | 0.25 | 0.26 abcd | |
| C22:5n6 | CTRL2 | 0.00-0.00 | 0.00 | 0.00 a | <0.001 |
| | PAP2 | 0.00-0.00 | 0.00 | 0.00 a | |
| | ALT2 | 0.00-0.00 | 0.00 | 0.00 a | |
| | CTRL3 | 0.00-0.00 | 0.00 | 0.00 a | |
| | PAP3 | 0.00-0.00 | 0.00 | 0.00 a | |
| | ALT3 | 0.00-0.00 | 0.00 | 0.00 a | |
| | CTRL4.5 | 0.10-0.11 | 0.11 | 0.11 bc | |
| | PAP4.5 | 0.10-0.12 | 0.11 | 0.11 bc | |
| | ALT4.5 | 0.11-0.21 | 0.21 | 0.18 c | |
| | CTRL6 | 0.09-0.11 | 0.10 | 0.10 bc | |
| | PAP6 | 0.08-0.09 | 0.09 | 0.09 b | |
| | ALT6 | 0.10-0.17 | 0.11 | 0.13 bc | |
| C22:5n3 | CTRL2 | 0.60-0.62 | 0.60 | 0.61 ab | <0.001 |
| | PAP2 | 1.15-1.21 | 1.17 | 1.18 d | |
| | ALT2 | 0.65-0.70 | 0.69 | 0.68 bc | |
| | CTRL3 | 0.56-0.57 | 0.57 | 0.57 a | |

| | | | | | |
|---|---|---|---|---|---|
| | PAP3 | 0.82-0.86 | 0.84 | 0.84 cd | |
| | ALT3 | 0.59-0.69 | 0.64 | 0.64 ab | |
| | CTRL4.5 | 0.75-1.09 | 0.88 | 0.90 cd | |
| | PAP4.5 | 0.80-1.05 | 0.93 | 0.93 cd | |
| | ALT4.5 | 0.62-0.67 | 0.62 | 0.64 ab | |
| | CTRL6 | 0.72-0.85 | 0.84 | 0.80 cd | |
| | PAP6 | 0.96-0.98 | 0.97 | 0.97 cd | |
| | ALT6 | 0.80-0.83 | 0.80 | 0.81 cd | |
| C22:6 c4,7,10,13,16,19 DHA / C22:6 n3 | CTRL2 | 5.14-5.44 | 5.28 | 5.29 h | <0.001 |
| | PAP2 | 5.74-5.85 | 5.75 | 5.78 i | |
| | ALT2 | 5.82-6.07 | 5.94 | 5.94 i | |
| | CTRL3 | 3.88-4.12 | 3.89 | 3.96 d | |
| | PAP3 | 4.96-5.03 | 5.03 | 5.01 gh | |
| | ALT3 | 4.91-5.04 | 4.97 | 4.97 fgh | |
| | CTRL4.5 | 4.36-4.38 | 4.38 | 4.37 de | |
| | PAP4.5 | 4.55-4.80 | 4.56 | 4.64 efg | |
| | ALT4.5 | 4.23-4.78 | 4.59 | 4.53 def | |
| | CTRL6 | 2.72-2.78 | 2.76 | 2.75 a | |
| | PAP6 | 2.93-3.00 | 2.97 | 2.97 b | |
| | ALT6 | 3.10-3.18 | 3.16 | 3.15 c | |

CTRL: Fish meal; PAP: Poultry and pig; ALT: Alternative protein; Least square means within a row having different letters differ significantly.a Significance values have been adjusted using the Bonferroni correction for multiple comparisons.

**Table S5**. Concentrations of the analyzed FAMEs (% of total fatty acids) in Sparus aurata in different fish feed formulations (CTRL, PAP, ALT) (n = 60).

| | Code | Min-Max | Median | Average * | p-value |
|---|---|---|---|---|---|
| C4:0 | CTRL | 0.00-0.00 | 0.00 | 0.00 | NA |
| | PAP | 0.00-0.00 | 0.00 | 0.00 | |
| | PAP | 0.00-0.00 | 0.00 | 0.00 | |
| C6:0 | CTRL | 0.00-0.00 | 0.00 | 0.00 | NA |
| | PAP | 0.00-0.00 | 0.00 | 0.00 | |
| | PAP | 0.00-0.00 | 0.00 | 0.00 | |
| C8:0 | CTRL | 0.06-0.22 | 0.10 | 0.11 a | 0.008 |
| | PAP | 0.07-0.34 | 0.15 | 0.17 b | |
| | PAP | 0.05-0.37 | 0.16 | 0.17 b | |
| C10:0 | CTRL | 0.01-0.02 | 0.01 | 0.01 a | <0.001 |
| | PAP | 0.01-0.05 | 0.01 | 0.02 a | |
| | PAP | 0.02-0.03 | 0.02 | 0.02 b | |
| C11:0 | CTRL | 0.00-0.00 | 0.00 | 0.00 | 0.368 |
| | PAP | 0.00-0.00 | 0.00 | 0.00 | |
| | PAP | 0.00-0.00 | 0.00 | 0.00 | |
| C12:0 | CTRL | 0.04-0.07 | 0.05 | 0.05 a | <0.001 |
| | PAP | 0.05-0.09 | 0.06 | 0.07 b | |
| | PAP | 0.89-1.27 | 1.08 | 1.10 c | |
| C14:0 | CTRL | 2.49-3.26 | 2.83 | 2.83 a | <0.001 |
| | PAP | 2.35-3.28 | 3.02 | 2.95 b | |
| | PAP | 2.92-3.67 | 3.32 | 3.33 c | |
| C15:0 iso | CTRL | 0.02-0.06 | 0.04 | 0.04 a | <0.001 |
| | PAP | 0.04-0.07 | 0.05 | 0.05 b | |
| | PAP | 0.02-0.05 | 0.04 | 0.04 a | |
| C15:0 anteiso | CTRL | 0.01-0.02 | 0.01 | 0.01 a | <0.001 |
| | PAP | 0.01-0.02 | 0.01 | 0.01 b | |
| | PAP | 0.01-0.04 | 0.01 | 0.01 b | |
| C14:1 | CTRL | 0.06-0.10 | 0.08 | 0.08 a | <0.001 |
| | PAP | 0.06-0.13 | 0.10 | 0.10 b | |
| | PAP | 0.07-0.16 | 0.10 | 0.11 b | |
| C15:0 | CTRL | 0.17-0.23 | 0.20 | 0.20 a | <0.001 |
| | PAP | 0.19-0.23 | 0.21 | 0.21 b | |
| | PAP | 0.33-0.42 | 0.35 | 0.35 c | |
| C16:0 iso | CTRL | 0.02-0.04 | 0.03 | 0.03 | 0.176 |
| | PAP | 0.01-0.05 | 0.02 | 0.03 | |
| | PAP | 0.02-0.05 | 0.03 | 0.03 | |
| C16:0 | CTRL | 15.06-17.51 | 16.22 | 16.21 | 0.351 |
| | PAP | 15.07-18.90 | 16.58 | 16.69 | |
| | PAP | 15.59-19.32 | 16.23 | 16.53 | |
| C17:0 iso | CTRL | 0.08-0.14 | 0.11 | 0.11 b | 0.004 |
| | PAP | 0.08-0.16 | 0.12 | 0.12 b | |

| | | | | | |
|---|---|---|---|---|---|
| | PAP | 0.07-0.17 | 0.10 | 0.10 a | |
| C16:1 c9 / C16:1 n7 | CTRL | 4.38-5.42 | 4.75 | 4.81 a | 0.008 |
| | PAP | 3.59-5.75 | 5.10 | 5.06 b | |
| | PAP | 3.37-5.65 | 4.76 | 4.71 a | |
| C17:0 | CTRL | 0.13-0.17 | 0.16 | 0.16 a | <0.001 |
| | PAP | 0.12-0.20 | 0.17 | 0.16 a | |
| | PAP | 0.14-0.20 | 0.19 | 0.18 b | |
| C16:3 n4 | CTRL | 0.09-0.17 | 0.12 | 0.12 a | <0.001 |
| | PAP | 0.12-0.26 | 0.16 | 0.17 b | |
| | PAP | 0.07-0.19 | 0.12 | 0.12 a | |
| C17:2 c9,10 | CTRL | 0.04-0.09 | 0.07 | 0.07 a | <0.001 |
| | PAP | 0.08-0.14 | 0.12 | 0.11 b | |
| | PAP | 0.18-0.26 | 0.23 | 0.22 c | |
| C16:2n4 | CTRL | 0.20-0.30 | 0.25 | 0.25 a | <0.001 |
| | PAP | 0.16-0.33 | 0.29 | 0.28 b | |
| | PAP | 0.18-0.28 | 0.24 | 0.23 a | |
| C18:0 | CTRL | 2.23-3.45 | 3.08 | 3.06 | 0.064 |
| | PAP | 2.74-3.97 | 3.04 | 3.11 | |
| | PAP | 2.29-3.35 | 2.83 | 2.88 | |
| C18:1 t9 | CTRL | 0.08-0.16 | 0.10 | 0.11 a | 0.003 |
| | PAP | 0.05-0.17 | 0.14 | 0.13 b | |
| | PAP | 0.06-0.15 | 0.12 | 0.12 a | |
| C18:1 c9 / C18:1 n9 | CTRL | 32.54-36.10 | 34.63 | 34.43 b | <0.001 |
| | PAP | 27.63-35.04 | 32.29 | 32.10 a | |
| | PAP | 28.83-34.53 | 32.26 | 32.11 a | |
| C18:1 n7 / C18:1 c11 | CTRL | 5.04-5.71 | 5.37 | 5.37 c | <0.001 |
| | PAP | 3.24-4.33 | 3.97 | 3.90 a | |
| | PAP | 2.71-4.82 | 4.29 | 4.20 b | |
| C19:0 | CTRL | 0.02-0.05 | 0.04 | 0.03 a | <0.001 |
| | PAP | 0.03-0.06 | 0.05 | 0.05 b | |
| | PAP | 0.02-0.06 | 0.04 | 0.04 a | |
| C18:2 t9,12 | CTRL | 0.01-0.01 | 0.01 | 0.01 a | <0.001 |
| | PAP | 0.01-0.06 | 0.02 | 0.02 c | |
| | PAP | 0.00-0.03 | 0.01 | 0.01 b | |
| C19:2 c9,10 | CTRL | 0.19-0.32 | 0.25 | 0.25 a | <0.001 |
| | PAP | 0.26-0.42 | 0.33 | 0.33 b | |
| | PAP | 0.20-0.38 | 0.28 | 0.28 a | |
| C18:2 c9,12 / C18:2 n6 | CTRL | 11.74-13.80 | 12.25 | 12.29 a | 0.023 |
| | PAP | 11.76-13.19 | 12.54 | 12.52 b | |
| | PAP | 11.65-12.93 | 12.54 | 12.45 b | |
| C18:2 n4 | CTRL | 0.10-0.18 | 0.14 | 0.14 | 0.194 |
| | PAP | 0.09-0.16 | 0.13 | 0.13 | |
| | PAP | 0.09-0.17 | 0.13 | 0.13 | |
| C20:0 | CTRL | 0.16-0.23 | 0.21 | 0.21 b | <0.001 |
| | PAP | 0.09-0.23 | 0.18 | 0.17 a | |

| | | | | | |
|---|---|---|---|---|---|
| | PAP | 0.11-0.20 | 0.17 | 0.17 a | |
| C18:3 c6,9,12 gamma / C18:3 n6 | CTRL | 0.08-0.22 | 0.13 | 0.13 a | <0.001 |
| | PAP | 0.14-0.33 | 0.23 | 0.22 c | |
| | PAP | 0.12-0.19 | 0.15 | 0.15 b | |
| C18:3 n4 | CTRL | 0.15-0.21 | 0.19 | 0.18 b | <0.001 |
| | PAP | 0.15-0.27 | 0.22 | 0.21 c | |
| | PAP | 0.12-0.19 | 0.16 | 0.16 a | |
| C20:1 c11 / C20:1 n9 | CTRL | 0.65-0.86 | 0.75 | 0.74 b | <0.001 |
| | PAP | 0.64-1.01 | 0.81 | 0.84 c | |
| | PAP | 0.46-0.72 | 0.60 | 0.62 a | |
| C18:3 c9,12,15 alpha / C18:3 n3 | CTRL | 2.61-3.21 | 2.86 | 2.86 b | <0.001 |
| | PAP | 2.11-2.86 | 2.65 | 2.58 a | |
| | PAP | 2.08-2.86 | 2.61 | 2.58 a | |
| C21:0 | CTRL | 0.01-0.03 | 0.02 | 0.02 | 0.200 |
| | PAP | 0.00-0.03 | 0.02 | 0.02 | |
| | PAP | 0.00-0.03 | 0.02 | 0.02 | |
| C18:4 n3 | CTRL | 0.36-0.55 | 0.47 | 0.47 b | <0.001 |
| | PAP | 0.42-0.98 | 0.57 | 0.58 c | |
| | PAP | 0.32-0.55 | 0.42 | 0.43 a | |
| C20:2 c11,14 | CTRL | 0.30-0.44 | 0.37 | 0.37 a | <0.001 |
| | PAP | 0.33-0.86 | 0.41 | 0.45 b | |
| | PAP | 0.13-0.44 | 0.36 | 0.35 a | |
| C22:0 | CTRL | 0.06-0.13 | 0.11 | 0.11 b | 0.022 |
| | PAP | 0.05-0.14 | 0.09 | 0.09 a | |
| | PAP | 0.03-0.16 | 0.09 | 0.09 a | |
| C20:3 c8,11,14 | CTRL | 0.12-0.27 | 0.20 | 0.20 a | <0.001 |
| | PAP | 0.21-0.58 | 0.24 | 0.28 b | |
| | PAP | 0.12-0.41 | 0.20 | 0.22 a | |
| C22:1 n11 | CTRL | 0.11-0.17 | 0.14 | 0.14 b | <0.001 |
| | PAP | 0.15-0.32 | 0.26 | 0.25 c | |
| | PAP | 0.05-0.17 | 0.09 | 0.09 a | |
| C22:1 c13 / C22:1 n9 | CTRL | 0.18-0.26 | 0.23 | 0.22 b | 0.003 |
| | PAP | 0.14-0.30 | 0.22 | 0.22 b | |
| | PAP | 0.10-0.26 | 0.18 | 0.18 a | |
| C20:3 c11,14,17 | CTRL | 0.04-0.19 | 0.12 | 0.12 a | <0.001 |
| | PAP | 0.09-0.17 | 0.14 | 0.14 b | |
| | PAP | 0.06-0.16 | 0.09 | 0.11 a | |
| C20:4 c5,8,11,14 / C20:4 n6 | CTRL | 0.47-0.87 | 0.54 | 0.60 a | 0.004 |
| | PAP | 0.44-1.05 | 0.75 | 0.75 b | |
| | PAP | 0.50-1.03 | 0.67 | 0.72 b | |
| C23:0 | CTRL | 0.01-0.04 | 0.02 | 0.02 a | <0.001 |
| | PAP | 0.02-0.05 | 0.03 | 0.03 b | |
| | PAP | 0.00-0.06 | 0.03 | 0.03 b | |
| C20:4 n3 | CTRL | 0.30-0.40 | 0.35 | 0.35 a | <0.001 |
| | PAP | 0.35-0.66 | 0.45 | 0.46 b | |

| | | | | | |
|---|---|---|---|---|---|
| | PAP | 0.30-0.54 | 0.36 | 0.36 a | |
| | CTRL | 0.06-0.12 | 0.08 | 0.08 | |
| C22:2 c13,16 | PAP | 0.02-0.17 | 0.10 | 0.09 | 0.297 |
| | PAP | 0.02-0.12 | 0.09 | 0.08 | |
| | CTRL | 0.00-0.00 | 0.00 | 0.00 | |
| C24:0 | PAP | 0.00-0.00 | 0.00 | 0.00 | NA |
| | PAP | 0.00-0.00 | 0.00 | 0.00 | |
| | CTRL | 4.37-5.74 | 4.84 | 4.97 | |
| C20:5 c5,8,11,14,17 EPA / C20:5 n3 | PAP | 4.38-7.16 | 5.32 | 5.37 | 0.083 |
| | PAP | 4.11-6.62 | 5.15 | 5.35 | |
| | CTRL | 0.17-0.29 | 0.22 | 0.23 | |
| C24:1 c15 / C24:1 n9 | PAP | 0.17-0.88 | 0.22 | 0.25 | 0.072 |
| | PAP | 0.10-0.27 | 0.20 | 0.20 | |
| | CTRL | 0.09-0.15 | 0.11 | 0.11 a | |
| C24:2 n6 | PAP | 0.07-0.25 | 0.11 | 0.12 ab | 0.022 |
| | PAP | 0.09-0.43 | 0.12 | 0.14 b | |
| | CTRL | 0.17-0.36 | 0.20 | 0.21 ab | |
| C21:5n3 | PAP | 0.14-0.46 | 0.22 | 0.23 b | 0.032 |
| | PAP | 0.12-0.30 | 0.19 | 0.19 a | |
| | CTRL | 0.10-0.19 | 0.14 | 0.15 a | |
| C22:5n6 | PAP | 0.10-0.41 | 0.16 | 0.17 b | <0.001 |
| | PAP | 0.14-0.26 | 0.20 | 0.20 c | |
| | CTRL | 1.87-2.10 | 2.05 | 2.05 | |
| C22:5n3 | PAP | 1.74-8.67 | 2.10 | 2.41 | 0.284 |
| | PAP | 1.78-3.00 | 2.01 | 2.08 | |
| | CTRL | 3.77-6.27 | 4.63 | 4.71 a | |
| C22:6 c4,7,10,13,16,19 DHA / C22:6 n3 | PAP | 4.28-7.65 | 5.61 | 5.56 b | <0.001 |
| | PAP | 4.33-8.00 | 5.65 | 5.99 b | |

CTRL: Fish meal-based diet; PAP: Processed Animal Protein-based diet; ALT: Alternative proteins-based diet; Least square means within a row having different letters differ significantly.
NA: Not available

**Table S6.** Nutritional recommendations, Health Claims and Nutritional Claims established in Regulation (EU) Nº 432/2012 and Regulation (EU) Nº 1924/2006.

| | | |
|---|---|---|
| | **Nutritional recommendations** | |
| Population groups | Recommendations | |
| Adults | The consumption of 250 mg of EPA+DHA per day is recommended | |
| Children | The consumption of 100 mg of DHA per day is recommended | |
| Pregnancy and lactation | The consumption of 250 mg of EPA+DHA per day plus 100 mg per day of DHA is recommended | |
| | **Health Claims** | |
| | Claims | Requirements |
| HC 1 | DHA helps maintain normal brain function | 40 mg DHA/100g<br>Beneficial effect: Daily intake of 250 mg of DHA |
| HC 2 | DHA helps maintain normal vision | 40 mg DHA/100g<br>Beneficial effect: Daily intake of 250 mg of DHA |
| HC 3 | DHA helps maintain normal triglyceride levels in the blood. | Daily intake: 2g EPA+DHA |
| HC 4 | DHA and EPA help maintain normal blood pressure. | Daily intake: 3g EPA+DHA |
| HC 5 | DHA and EPA help maintain normal triglyceride levels in the blood. | Daily intake: 2g EPA+DHA |
| HC 6 | EPA and DHA help maintain the normal function of the heart. | Daily intake: 250 mg DHA+EPA |
| | **Nutritional Claims** | |
| | Claims | Requirements |
| NC 1 | Source of omega-3 fatty acids | 0.3 g of alpha-linolenic acid/ 100 g<br>40 mg EPA+DHA/100g |
| NC 2 | High content of omega-3 fatty acids | 0.6 g of alpha-linolenic acid/ 100 g<br>80 mg EPA+DHA/100g |
| NC 3 | High content of monounsaturated fats | 45% of the fatty acids are monounsaturated and more than 20% of the product's total energy value |
| NC 4 | High content of polyunsaturated fats | 45% of the fatty acids are polyunsaturated and more than 20% of the product's total energy value. |
| NC 5 | High content of unsaturated fats | 70% of the fatty acids are unsaturated and more than 20% of the product's total energy value |

*HC: Health Claim; NC: Nutritional Claim
